# Care patterns and Traditional Chinese Medicine constitution as factors of depression and anxiety in patients with systemic sclerosis: A cross-sectional study during the COVID-19 pandemic

**DOI:** 10.3389/fnint.2023.1052683

**Published:** 2023-02-14

**Authors:** Qi Kong, Li-Ming Chen, Zong-Hao Dai, Yun-Zhe Tang, Yu-Yang Zhou, Wen-Zhen Tu, Yin-Huan Zhao, Jia-Qian Zhang

**Affiliations:** ^1^Scientific Innovation Volunteer Team of Rare Diseases, Shanghai TCM-Integrated Hospital, Shanghai University of Traditional Chinese Medicine, Shanghai, China; ^2^Putuo Hospital, Shanghai University of Traditional Chinese Medicine, Shanghai, China; ^3^Yueyang Hospital of Integrated Traditional Chinese and Western Medicine, Shanghai University of Traditional Chinese Medicine, Shanghai, China; ^4^Department of Vascular Diseases, Shanghai TCM-Integrated Hospital, Shanghai University of Traditional Chinese Medicine, Shanghai, China; ^5^School of Public Health, Shanghai University of Traditional Chinese Medicine, Shanghai, China; ^6^Department of Rheumatology, Shanghai TCM-Integrated Hospital, Shanghai University of Traditional Chinese Medicine, Shanghai, China

**Keywords:** systemic sclerosis, care patterns, TCM constitution, depression, anxiety, Scleroderma, suicidal ideation

## Abstract

**Objective:**

Care patterns and Traditional Chinese Medicine (TCM) constitution affects the emotion and health of patients with systemic sclerosis (SSc) while the prevalence of COVID-19 may aggravate such patients’ emotion and health. We investigated the depression and anxiety levels of patients with SSc during the pandemic to identify the correlation between care patterns, TCM constitution, and patients’ emotion.

**Materials and methods:**

This was a cross-sectional study. Patients with SSc and healthy individuals were surveyed using the patient health questionnaire-9, generalized anxiety disorder-7, and constitution in Chinese medicine questionnaire and a modified care pattern questionnaire. Factors correlated with depression and anxiety were screened using univariate and multivariate logistic regression analyses.

**Results:**

A total of 273 patients with SSc and 111 healthy individuals were included in the analysis. The proportion of patients with SSc who were depressed was 74.36%, who had anxiety was 51.65%, and who experienced disease progression during the pandemic was 36.99%. The proportion of income reduction in the online group (56.19%) was higher than that in the hospital group (33.33%) (*P* = 0.001). Qi-deficiency [adjusted odds ratio (OR) = 2.250] and Qi-stagnation (adjusted OR = 3.824) constitutions were significantly associated with depression. Remote work during the outbreak (adjusted OR = 1.920), decrease in income (adjusted OR = 3.556), and disease progression (*P* = 0.030) were associated with the occurrence of depression.

**Conclusion:**

Chinese patients with SSc have a high prevalence of depression and anxiety. The COVID-19 pandemic has changed the care patterns of Chinese patients with SSc, and work, income, disease progression, and change of medications were correlates of depression or anxiety in patients with SSc. Qi-stagnation and Qi-deficiency constitutions were associated with depression, and Qi-stagnation constitution was associated with anxiety in patients with SSc.

**Trial registration:**

http://www.chictr.org.cn/showproj.aspx?proj=62301, identifier ChiCTR2000038796.

## 1. Introduction

Systemic sclerosis (SSc) is a complex and rare autoimmune disease that often begins manifesting in the hands. Patients with SSc present with skin fibrosis and Raynaud’s phenomenon in the early stages and fibrosis, which can involve the facial skin and organs (most commonly the lungs) in the middle and late stages, seriously affecting the survival quality of patients and causing a high disability rate (Almeida et al., 2015; Denton and Khanna, 2017). Patients with SSc suffer from disease flare-ups. The flare-ups accelerate disease aggravation and spread inflammation to systemic organs, leading to increasing risk of hospitalization and surgery. Therefore, patients need long-term immunosuppressive medications to control inflammation (Denton and Khanna, 2017; Waszczykowski et al., 2021). Out-of-hospital health management and regular follow-up visits are also necessary to keep the disease stable. These are key elements of the care patterns of SSc and other similar inflammatory chronic diseases. Depression and anxiety are common symptoms in patients with chronic inflammatory diseases, and has been widely re reported in SSc (Amaral et al., 2013; Kwakkenbos et al., 2015; Pagkopoulou et al., 2019). These emotional problems result from social stress and physical factors and may lead to psychiatric illness, decreased compliance, and life quality deterioration.

During the COVID-19 pandemic, patients with chronic diseases were forced to change their lifestyle and care patterns. Meanwhile, their income reduced due to work restrictions. These challenged the management of their disease and mood. Data on inflammatory bowel disease (Chen et al., 2020) and osteoporosis (Oliveira et al., 2022) show that after care patterns were changed by the pandemic, patients experience changes in visiting physicians at the health center to telemedicine and erratic medication use. These problems also imposed psychological stress on the patients (Qiu et al., 2020). Thombs et al. (2020) reported results on SSc cohorts from four countries and found increased anxiety in the UK and US cohorts and non-significant changes in the France and Canada cohorts post-COVID-19 pandemic compared with pre-COVID-19 pandemic. Furthermore, no significant increase in depression was found. These changes may be attributed to sundry pandemic control mechanisms in different countries that affected the lives of patients with SS through care patterns.

Traditional Chinese Medicine (TCM) is a traditional system of complementary alternative medicine, which includes treatments such as acupuncture, herbal medicine, Tai Chi, etc. TCM constitution is a modern branch of the TCM theory. It classifies a person’s physical state into nine categories by quantifying symptoms: one normal constitution category and eight deviant constitution categories. Health care strategies and medication references accompany the description of each constitution, following the TCM theories (Li et al., 2021). Currently, the TCM constitution theory and its scale tool are widely used in the field of health care in China (including mainland China, Macau, Hong Kong, and Taiwan) as well as in Japan and Korea and have been extended to the study of various diseases such as cancer, hypertension, metabolic syndrome, and multiple sclerosis (Liang et al., 2020). A study showed that yang and yin deficiency constitutions are associated with a higher risk of depression in adult female populations (Chen et al., 2021). As patients with SSc are predominantly female, the TCM constitution may act as an intrinsic factor influencing emotion.

In this study, we reported depression, anxiety, and TCM constitution in patients with SSc in mainland China. Further, we investigated the correlation between care pattern, TCM constitution factors, and depression-anxiety occurrence in patients with SSc during the COVID-19 pandemic.

## 2. Materials and methods

### 2.1. Basic information

This was a cross-sectional study conducted from October to November 2020. Continuous sampling method was used to conduct data collect data both online and offline. Regarding the online data collection, we posted the recruitment information through the China Organization for Scleroderma (also known as “Purple Shell”), one of the largest online communities for patients with SSc in mainland China. After the participants responded to the recruitment information, volunteers from the organization first reviewed whether the participants met the inclusion criteria. These volunteers had received the necessary training in the organization. Wenjuanxing (WJX) is an online survey platform that is commonly used within the Chinese online community. All questionnaires were distributed and collected through WJX after it was published in the online community, and the patients filled them voluntarily. Patients who completed the questionnaire received a small financial token of appreciation. According to the questionnaire platform settings, patients needed to complete all the questions before they could submit the questionnaire. Regarding the offline questionnaires, all the patients who patients who completed the questionnaire were diagnosed by a specialist were from outpatient or inpatient of rheumatology departments at Shanghai Hospital of Integrative Medicine, Shanghai University of Traditional Chinese Medicine. Online and offline data were cross-checked to exclude duplicate survey responses. To compare the impact of the pandemic between different populations, data from the healthy population were collected in this study. We randomly selected healthy volunteers from the streets of Shanghai, Lianyungang, and Kunming to fill the questionnaires. A wider range of population could be represented in these three cities, which differ in their level of development. Members of the research team traveled to the local area to conduct a street survey. After quality control, the participants who met the inclusion criteria were included in the study. Data collectors and volunteers confirmed that the patients understood the survey information accurately and helped patients understand the questionnaire content if necessary. Due to the rarity of patients with SSc and the complexity of the study design, we collected the largest possible sample within a certain time frame to meet the requirement of 10 events per variable in the subsequent regression analysis (Vittinghoff and McCulloch, 2007). This study is reported according to the Strengthening the Reporting of Observational Studies in Epidemiology (STROBE) guidelines (Vandenbroucke et al., 2007).

### 2.2. Inclusion and exclusion criteria

Participants were Chinese mainland residents aged 18–75 years and were required to be capable of thinking and expressing themselves clearly, have normal verbal and sensory responses, and understand the specifics and implications of this study. Patients participating online needed to be patient members of the China Organization for Scleroderma. Volunteers from Purple Shell selected and called back some of the online participants to confirm their responses. Online patients should have been diagnosed with SSc for more than 6 months by a relevant specialty in a third-grade class-A hospital (similar to a regional medical center in North America). Patients in the hospital were required to meet the 2013 American College of Rheumatology and European League Against Rheumatism classification criteria for SSc (van den Hoogen et al., 2013) and should have been diagnosed with SSc for more than 6 months. Patients with a history of psychiatric diagnosis, stroke, severe trauma, tumor, and other major secondary non-SSc diseases were excluded from this study. Healthy participants were required to confirm if they had history of mental illness diagnosis, stroke, serious trauma, tumors, and other chronic medical conditions requiring long-term treatment for more than 6 months. In the online survey, there was no missing data because of the pre-set rules for questionnaire submission. The hospital group questionnaires with significant missing data were excluded from this study.

## 3. Measuring tools

### 3.1. Care patterns

Chen et al. (2020) conducted a survey on personal health care among patients with Crohn’s disease in mainland China during the COVID-19 epidemic in China. An 18-item questionnaire, which asked patients about dimensions including employment, emotional, and disease status, was used for the survey. Crohn’s disease shares many similarities with SSc in terms of disease characteristics and social factors. We modified the questionnaire for the patients with SSc in this study based on the questionnaire used by Chen et al. The questionnaire was pre-tested on a small scale with Purple Shell community administrators before the formal testing to confirm accurate understanding. After the questionnaires were collected, preliminary calculations revealed that the questionnaires had good reliability. The emotion part of the questionnaire had good criterion validity (Supplementary Table 7). The original language of the questionnaire was Chinese, and its English translation is provided in the Supplementary Table 1.

### 3.2. TCM constitution

We used the Constitution in Chinese Medicine Questionnaire (CCMQ) designed by Wang Qi, which has good reliability and validity (Wong et al., 2013; Jing et al., 2015; Zhu et al., 2019) (Supplementary Table 6). The questionnaire contained 60 items and was divided into nine dimensions: Balanced, Yang-deficiency, Yin-deficiency, Qi-deficiency, Qi-stagnation, phlegm-dampness, Dampness-heat, Blood-stasis, and Inherited special constitution (Supplementary Table 5; Chen, 2021). One can have more than one constitution or composite constitutions. The balanced constitution is the closest one to good health. A person who is judged to have a balanced constitution will not be defined as having the other biased constitutions. The constitution of a participant was judged based on the score in each dimension. If a participant had a balanced constitution score ≥ 60 and all other constitution scores < 30, that participant was considered as having a balanced constitution. If the score of the other dimension was ≥ 40, the participant was considered having the constitution represented by that dimension. Biased constitution scores of 30–39 were not included in the analysis. Particularly, a major constitution was defined as a balanced constitution or the highest score on the corresponding dimension other than the balanced constitution. One may have two or more major constitutions. The detailed calculation process is shown in Supplementary Figure 1.

### 3.3. Depression, anxiety, and suicidal ideation

The Patient Health Questionnaire-9 (PHQ-9) and Generalized Anxiety Disorder-7 (GAD-7) were first used by Spitzer and Kroenke (Spitzer et al., 1999), allowing for self-assessment by survey respondents. The Chinese version of the two questionnaires were considered to have good reliability and validity (Wang et al., 2014, 2018). PHQ-9 and GAD-7 are now widely used in medical screening for depression and anxiety. The two questionnaires describe 16 scenarios with four scores (0–3) based on the frequency of each scenario. The PHQ-9 has three generic cut-off scores of 5,10, and 15 corresponding to mild, moderate, and severe depression. For the GAD-7, the scores are 5, 10, and 14. To screen for depressive symptoms in chronic disease more accurately, we used the mild cut-off scores of both scales as the main outcome (Wańkowicz et al., 2020). The last scenario of the PHQ-9 was considered as a screening item for suicidal ideation (Razykov et al., 2013).

### 3.4. Data statistics

SPSS 24.0 (IBM, New York, NY, USA) was used to analyze the data. Descriptive statistics were expressed as median and interquartile range [M(IQR)]. Comparisons between groups of patients and healthy participants were performed using the Wilcoxon rank sum test. Multiple group comparisons were performed using the Kruskal-Wallis *H* test, and *post hoc* analysis between two groups was performed using the Bonferroni method. The chi-square test was used for comparison of two categorical data. Cronbach’s test and Pearson’s test were used to test the reliability and validity of the questionnaire. Correlation factors were analyzed using univariate and multivariate logistic regression analyses to adjust for confounding factors. Factors with *P* < 0.10 in the univariate regression analysis were included in the multivariate regression analysis. The information underlying the two models is presented together in the results to reflect the sensitivity of the conclusions. TCM constitution, major TCM constitution, and care patterns were screened separately to avoid the occurrence of co-linearity. The significance level was set as *α* = 0.05.

## 4. Results

### 4.1. Demographics

**FIGURE 1:**
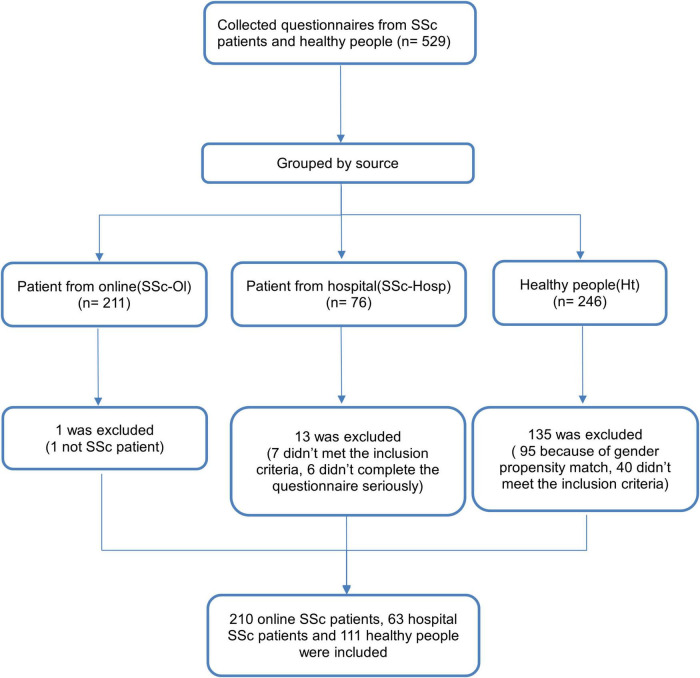
Study process.

A total of 273 patients with SSc were included in this study, of which 210 were online while 63 were from the outpatient or inpatient unit of rheumatology departments at Shanghai TCM-Integrated Hospital. There were 111 healthy participants (Figure 1). The morbidity characteristics of patients with SSc had a significant gender deviation (Hughes et al., 2020). Among the study participants, female patients accounted for 90.48%. To account for the influence of gender on the results, we randomly selected healthy volunteer participants according to the same sex ratio. For every nine healthy female volunteers included, we randomly selected the questionnaire of one eligible male to be included in the final data analysis. The median age of both the patient and the healthy groups was 45 years each, which was not significantly different. Height, weight, and body mass index were significantly lower in the patient group than in the healthy group. There were no significant differences in any of the demographic data between the patients from different sources (Table 1).

**TABLE 1:** Demographic data.

	SSc-Pt (*n* = 273)	SSc-Ol (*n* = 210)	SSc-Hosp (*n* = 63)	Ht (*n* = 111)	*P* ^a^
Gender (female), n (%)	247 (90.48)	191 (91.00)	56 (88.90)	101 (91.00)	0.875
Age (years), M (IQR)	45.00 (7.00)	44.00 (18.00)	47.00 (18.00)	45.00 (14.00)	0.578
Height (cm), M (IQR)	160.00 (8.00)^b^	160.00 (8.00)^b^	160.00 (8.00)	161.00 (7.00)	0.007
Weight (kg), M (IQR)	55.00 (12.25)^c^	55.00 (12.63)^b^	54.00 (12.50)^c^	60.00 (14.00)	<0.001
BMI (kg/m^2^), M (IQR)	20.96 (4.41)^c^	21.33 (4.19)^b^	20.45 (3.87)^b^	22.15 (4.74)	<0.001
Durations (years), M (IQR)	6.00 (7.00)	6.00 (7.00)	6.00 (9.00)	-	-

SSc-Pt, systemic sclerosis patients; SSc-Ol, online systemic sclerosis patients; SSc-Hosp, hospital systemic sclerosis patients; Ht, healthy person; BMI, body mass index.

^a^Using Kruskal-Wallis *H* test, the corresponding numerical statistics of online patients, hospital patients, and healthy individuals for *P*-value.

^b^Using Kruskal-Wallis *H* test, the corresponding numerical statistics with healthy individuals adjusted by Bonferroni correction method with *P* < 0.01.

^c^Using Kruskal-Wallis *H* test, the corresponding numerical statistics with healthy individuals adjusted by Bonferroni correction method with *P* < 0.001.

### 4.2. Care patterns

**FIGURE 2:**
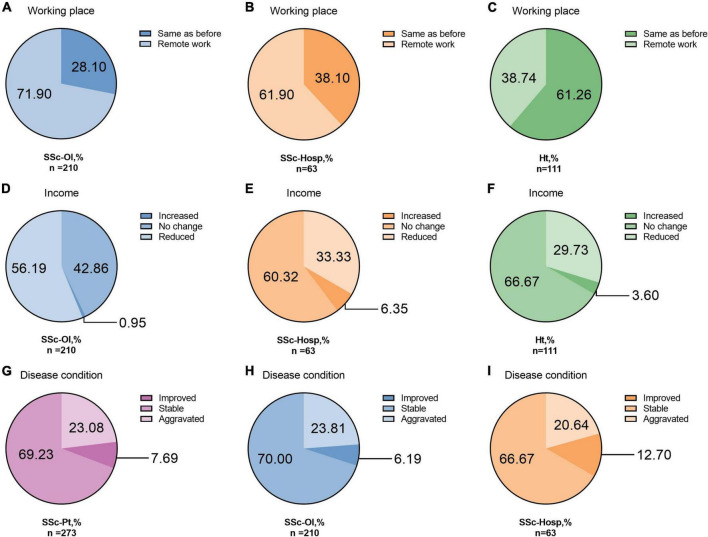
Work place, income and disease condition. **(A)** Working place of online systemic sclerosis patients. **(B)** Working place of hospital systemic sclerosis patients. **(C)** Working place of healthy person. **(D)** Income of online systemic sclerosis patients. **(E)** Income of hospital systemic sclerosis patients. **(F)** Income of healthy person. **(G)** Disease condition of all systemic sclerosis patients. **(H)** Disease condition of online systemic sclerosis patients. **(I)** Disease condition of hospital systemic sclerosis patients. SSc-Pt, systemic sclerosis patients; SSc-Ol, online systemic sclerosis patients; SSc-Hosp, hospital systemic sclerosis patients; Ht, healthy person.

Regarding care patterns, 69.60% of patients worked remotely during the pandemic, and 50.92% experienced a decrease in income; both were higher than the proportions in the healthy group (38.74 and 29.73%, respectively). A higher percentage of patients in the online group (56.19%) had a lower income than that of those in the hospital group (33.33%) (*P* = 0.001). Regarding the patient-evaluated disease status, 36.99% of the patients had an aggravated disease during the pandemic and 23.81% were afraid to seek medical support outside their home, which was not significantly different between the hospital and online groups (*P* = 0.927 and 0.060, respectively) (Figure 2).

34 patients (12.45%) accessed telemedicine during the pandemic. Most of the patients with SSc received medical support through Apps as well as from specialist physicians. The majority of patients used the same medication during the pandemic as those used before the pandemic (191, 69.96%). Disease status was the main reason for the change in medication use. More than half of the total surveyed patients (50.18%) obtained their oral medications in the hospital during the COVID-19 outbreak, and another proportion of patients (32.23%) obtained their medications online. Notably, patient organizations also played a role in medication delivery, providing oral medications to 20.51% of the patients. As of October 2020, 23.08% of the patients felt the disease had aggravated, and 7.69% of the patients were afraid to seek medical support, a significant decrease from that before the pandemic (*P* < 0.001). After the pandemic, more than half of the patients (60.07%) expect to increase the number of medical visits (Supplementary Table 2).

### 4.3. TCM constitution

**FIGURE 3:**
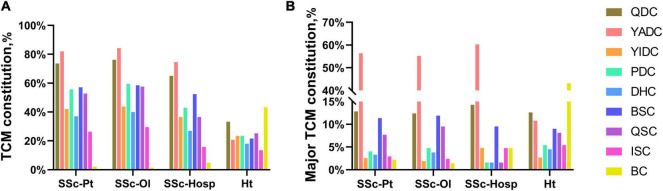
Traditional Chinese Medicine (TCM) constitution rate. **(A)** TCM constitution. **(B)** Major TCM constitution. SSc-Pt, systemic sclerosis patients; SSc-Ol, online systemic sclerosis patients; SSc-Hosp, hospital systemic sclerosis patients; Ht, healthy person; BC, balanced constitution; YADC, Yang-deficiency constitution; YIDC, Yin-deficiency constitution; QDC, Qi-deficiency constitution; QSC, Qi-stagnation constitution; PDC, phlegm-dampness constitution; DHC, Dampness-heat constitution; BSC, blood-stasis constitution; ISC, inherited special constitution; TCM constitution, Traditional Chinese Medicine constitution.

Yang-deficiency constitution was the most common TCM constitution characteristic of the patients with SSc, accounting for 82.05% of the total participants. Meanwhile, Qi-deficiency and blood-stasis constitutions were also more widespread, accounting for 73.63 and 57.14%, respectively. Among the major constitutions, these three also accounted respectively for more than 10% of all the TCM constitutions of the patients. The distribution of constitutions was generally similar in patients from different populations. In the healthy group, each constitution was more evenly distributed, with the balanced constitution being the dominant one, accounting for 43.24%. The balanced constitution in the patient group was only 2.20% (Figure 3 and Supplementary Table 3).

### 4.4. Depression, anxiety, suicidal ideation, and correlation factors

**FIGURE 4:**
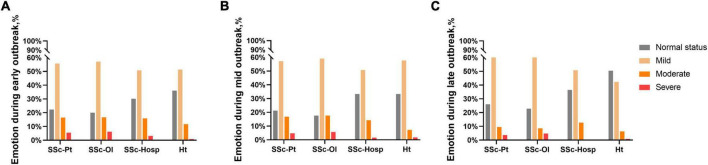
Emotion related result. **(A)** Emotion during early outbreak. **(B)** Emotion during mid outbreak. **(C)** Emotion during late outbreak. SSc-Pt, systemic sclerosis patients; SSc-Ol, online systemic sclerosis patients; SSc-Hosp, hospital systemic sclerosis patients; Ht, healthy person.

In the care pattern questionnaire, we investigated the effect of the pandemic on participants’ emotions. Throughout the pandemic, patients with SSc had worse emotions compared to those of the healthy group. Further, the online group was in worse emotional condition than that of the hospital group; however, the difference was not significant (*P* = 0.522). At the time of the survey, most healthy individuals (92.79%) had returned to normal emotional state, while less than 30% of the patients with SSc had normal emotional state. The healthy participants had better emotional regulation than that observed among the patients with SSc (Figure 4).

**FIGURE 5:**
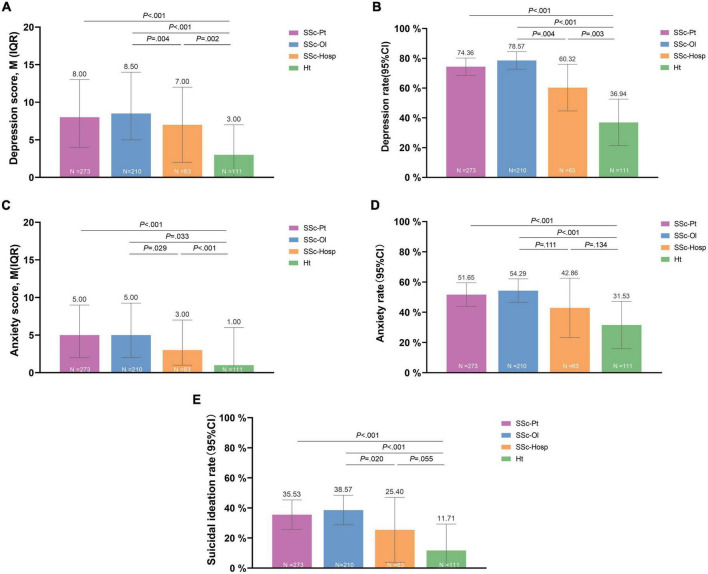
Depression, anxiety, and suicidal ideation. **(A)** Depression score. **(B)** Depression rate. **(C)** Anxiety score. **(D)** Anxiety rate. **(E)** Suicial ideation rate. SSc-Pt, systemic sclerosis patients; SSc-Ol, online systemic sclerosis patients; SSc-Hosp, hospital systemic sclerosis patients; Ht, healthy person.

We used the PHQ-9 and GAD-7 to screen depression and anxiety in the SSc and healthy groups. At the mild cut-off value, 74.36% of patients with SSc had depression and 51.65% had anxiety, both of which were significantly higher than that in the healthy group (*P* < 0.001). At the moderate cut-off value, 43.59% of the patients with SSc had depression and 23.08% had anxiety while at the high cut-off value, the proportion was 21.61% for depression and 12.82% for anxiety (Supplementary Figure 2). The online group had significantly higher depression scores, depression rates, and anxiety scores than those had by the hospital group. The proportion of patients with SSc who had suicidal ideation was 35.53% and that of the healthy group was 11.71% (*P* < 0.001; Figure 5).

We analyzed and identified significant factors of depression, anxiety, and suicidal ideation using multivariate logistic regression (Table 2). The complete table is provided in the supplementary material (Supplementary Table 4). We found that Qi-deficiency constitution [adjusted odds ratio (OR) = 2.250] and Qi-stagnation constitution (adjusted OR = 3.824) were significantly associated with depression in patients with SSc. Dampness-heat constitution was associated with a decrease in depression (adjusted OR = 0.160). In the care patterns, remote work during the outbreak (adjusted OR = 1.920) and disease progression (adjusted OR = 3.556) were both correlates of depression. Basically, the online group was similar to the hospital group. Phlegm-dampness constitution was independently associated with depression in the hospital group (adjusted OR = 7.537) (Table 2A). The main constitution for anxiety in patients with SSc was Qi-stagnation constitution (adjusted OR = 4.567). Partial changes in medication (adjusted OR = 2.884) and total changes or discontinuation of medication (adjusted OR = 2.608) were significantly associated with the occurrence of anxiety, except for those who experienced changes in work status. In the healthy group, the Yang-deficiency constitution (adjusted OR = 3.818) was associated with anxiety. Healthy individuals with balanced constitution as their major constitution had an associated lower risk of anxiety or depression (adjusted OR = 0.124, 0.171; Table 2B). Factors for suicidal ideation were similar to those for anxiety in patients with SSc. Provision of oral medications by patient organizations was associated with suicidal ideation in the hospital group (adjusted OR = 12.124). Yang-deficiency constitution (adjusted OR = 6.839) and having Yang-deficiency or dampness-heat constitutions as a major constitution (adjusted OR = 5.145, 14.352) in the healthy group were significantly associated with the risk of suicidal ideation. Change in income during the pandemic was also significantly associated with suicidal ideation in the healthy group (Table 2C).

**TABLE 2A:** Regression analysis of related factors of depression, anxiety, and suicidal ideation (Depression).

Source	Question	Item	*P*	OR (95% CI)	Adjusted *P*	Adjusted OR (95% CI)
SSc-Pt		Qi-stagnation constitution	< 0.001	7.978 (4.092, 15.551)	< 0.001	3.824 (1.799, 8.126)
SSc-Pt		Qi-deficiency constitution	< 0.001	6.481 (3.553, 11.823)	0.025	2.250 (1.109, 4.562)
SSc-Pt		Dampness-heat constitution (main)	0.011	0.160 (0.039, 0.658)	0.011	0.160 (0.039, 0.658)
SSc-Pt	1	Change in working condition during the outbreak (Dec 2019–Feb 2020)	0.004	2.308 (1.309, 4.070)	0.041	1.920 (1.027, 3.589)
SSc-Pt	7	Disease progress during the outbreak (Dec 2019–Feb 2020)	< 0.001	-	0.014	-
	7B	Stable (#dummy variable)	-	-	-	-
	7A	Improved	0.565	0.672 (0.174, 2.602)	0.743	0.761 (0.149, 3.888)
	7C	Aggravated	< 0.001	5.497 (2.580, 11.712)	0.005	3.556 (1.466, 8.626)
SSc-Ol		Qi-deficiency constitution	< 0.001	6.110 (2.974, 12.552)	0.046	2.421 (1.016, 5.767)
SSc-Ol		Qi-stagnation constitution	< 0.001	8.453 (3.797, 18.816)	< 0.001	5.151 (2.114, 12.551)
SSc-Ol		Dampness-heat constitution (main)	0.011	0.148 (0.034, 0.646)	0.011	0.148 (0.034, 0.646)
SSc-Hosp		Phlegm-dampness constitution	< 0.001	22.115 (4.497, 108.749)	0.032	7.537 (1.187, 47.851)
SSc-Hosp	1	Change in working condition during the outbreak (Dec 2019–Feb 2020)	0.005	4.833 (1.617, 14.451)	0.007	4.873 (1.545, 15.371)
Ht		Balanced constitution (main)	< 0.001	0.146 (0.057, 0.374)	< 0.001	0.171 (0.065, 0.447)

**TABLE 2B:** Anxiety.

Source	Question	Item	*P*	OR (95% CI)	Adjusted *P*	Adjusted OR (95% CI)
SSc-Pt		Qi-stagnation constitution	< 0.001	6.465 (3.814, 10.959)	< 0.001	4.567 (2.504, 8.328)
SSc-Pt		Qi-stagnation constitution (main)	0.025	3.251 (1.156, 9.144)	0.025	3.251 (1.156, 9.144)
SSc-Pt	11	Change in SSc medications during the outbreak (Dec 2019–Feb 2020)	< 0.001	-	0.006	-
	11B	No change (#dummy variable)	-	-	-	-
	11A	Change in few/Most medications	< 0.001	3.717 (2.042, 6.763)	0.002	2.884 (1.452, 5.729)
	11C	Change in/Discontinuation of all medications	0.063	3.621 (0.932, 14.076)	0.192	2.608 (0.619, 10.991)
SSc-Ol		Qi-stagnation constitution	< 0.001	6.462 (3.520, 11.860)	< 0.001	4.885 (2.470, 9.660)
SSc-Ol	11	Change in SSc medications during the outbreak (Dec 2019–Feb 2020)	0.002	-	0.011	-
	11B	No change (#dummy variable)	-	-	-	-
	11A	Change in few/Most medications	0.001	3.223 (1.641, 6.330)	0.006	2.700 (1.335, 5.458)
	11C	Change in/Discontinuation of all medications	0.129	3.537 (0.691, 18.110)	0.152	3.344 (0.641, 17.444)
SSc-Hosp	1	Change in working condition during the outbreak (Dec 2019–Feb 2020)	0.008	4.918 (1.525, 15.859)	0.008	5.882 (1.581, 21.888)
	11	Change in SSc medications during the outbreak (Dec 2019–Feb 2020)	0.018	-	0.020	-
	11B	No change (#dummy variable)	-	-	-	-
	11A	Change in few/Most medications	0.007	6.089 (1.648, 22.497)	0.007	7.348 (1.715, 31.475)
	11C	Change in/Discontinuation of all medications	0.240	4.429 (0.370, 52.990)	0.242	5.048 (0.335, 76.084)
Ht		Yang-deficiency constitution	< 0.001	11.019 (3.798, 31.967)	0.034	3.818 (1.104, 13.199)
Ht		Balanced constitution (main)	< 0.001	0.094 (0.030, 0.292)	0.001	0.124 (0.037, 0.412)

**TABLE 2C:** Suicidal ideation.

Source	Question	Item	*P*	OR (95% CI)	Adjusted *P*	Adjusted OR (95% CI)
SSc-Pt		Qi-deficiency constitution	< 0.001	4.732 (2.294, 9.758)	0.036	2.471 (1.061, 5.757)
SSc-Pt		Qi-stagnation constitution	< 0.001	4.872 (2.792, 8.502)	< 0.001	3.676 (1.937, 6.975)
SSc-Pt		Qi-stagnation constitution (main)	0.037	2.620 (1.062, 6.462)	0.037	2.620 (1.062, 6.462)
SSc-Pt	11	Change in SSc medications during the outbreak (Dec 2019–Feb 2020)	< 0.001	-	0.009	
	11B	No change (#dummy variable)	-	-	-	
	11A	Change in few/Most medications	< 0.001	3.976 (2.245, 7.041)	0.002	2.951 (1.477, 5.895)
	11C	Change in/Discontinuation of all medications	0.487	1.569 (0.441, 5.583)	0.635	1.436 (0.323, 6.393)
SSc-Ol		Qi-stagnation constitution	< 0.001	4.284 (2.286, 8.031)	0.001	3.059 (1.537, 6.092)
SSc-Ol		Qi-stagnation constitution (main)	0.044	2.630 (1.025, 6.749)	0.044	2.630 (1.025, 6.749)
SSc-Ol	11	Change in SSc medications during the outbreak (Dec 2019–Feb 2020)	0.001	-	0.006	-
	11B	No change (#dummy variable)	-	-	-	-
	11A	Change in few/Most medications	< 0.001	3.583 (1.885, 6.810)	0.001	3.062 (1.540, 6.087)
	11C	Change in/Discontinuation of all medications	0.661	1.391 (0.318, 6.076)	0.642	1.432 (0.315, 6.510)
SSc-Hosp	13E	From patient associations	0.029	7.500 (1.224, 45.961)	0.025	12.124 (1.375, 106.912)
Ht		Yang-deficiency constitution	0.001	8.853 (2.548, 30.757)	0.005	6.839 (1.801, 25.975)
Ht		Yang-deficiency constitution (main)	0.022	5.000 (1.255, 19.915)	0.029	5.145 (1.179, 22.456)
Ht		Dampness-heat constitution (main)	0.006	14.40 (2.145, 96.667)	0.008	14.852 (2.045, 107.864)
Ht	2	Income changed during the outbreak (Dec 2019–Feb 2020)	0.012	-	-	-
	2B	No change (#dummy variable)	-	-	-	-
	2A	Increased	0.011	17.500 (1.932, 158.538)	-	-
	2C	Reduced	0.020	4.712 (1.273, 17.433)	-	-

SSc-Pt, systemic sclerosis patients; SSc-Ol, online systemic sclerosis patients; SSc-Hosp, hospital systemic sclerosis patients; Ht, healthy person.

## 5. Discussion

Emotional problems in patients with SSc have been studied to date. According to an earlier systematic review, the prevalence of depressive symptoms in patients with SSc was 51–65% (Thombs et al., 2007). Iranian data showed a prevalence of 68.4 and 23.6% for depressive and anxiety symptoms in patients with SSc, respectively (Faezi et al., 2017). Besides, in a recently published cohort study, the rates of anxiety, depression, mixed anxiety-depressive disorder, and distress in patients with SSc were 32.2, 25.9, 18.5, and 49.5%, respectively (Jiang et al., 2022). An European cohort study demonstrated that COVID-19 increased anxiety symptoms in a group of patients with SSc (Thombs et al., 2020). Among the symptoms associated with SSc, pain due to Raynaud’s phenomenon, arthralgia, finger ulcers, and dysphagia may be associated with depression (Ostojic et al., 2019). Anxiety is associated with life quality in patients with SSc (Sierakowska et al., 2019). To our knowledge, this study is the first cross-sectional study on depression and anxiety in the SSc population in China. In this study, we screened both extrinsic and intrinsic factors of anxiety and depression in the SSc population. We considered the care patterns as the extrinsic effects of COVID-19 on patients with SSc and the TCM constitution as the intrinsic factors.

Care patterns for chronic diseases during the COVID-19 pandemic underwent multiple challenges (Mafi et al., 2022). These patterns included patients’ work, income, medical treatment, sources of oral medications, and disease progression (Chen et al., 2020). The way of life and work, environmental factors, and medical and health services, as part of the care patterns, are closely related to physical and mental health (Orlandi et al., 2020). Personal travel was restricted based on local policies or the fear of infection. Cases of COVID-19 also crowded out local medical resources. This undoubtedly affected regular medical visits for patients with chronic diseases, and subsequently weakened the management of their disease (Yildirim et al., 2021). Meanwhile, the pandemic has reduced regional economic activity, with a subsequent impact on individual work and income (Batterham et al., 2021). Patients with chronic diseases compared with healthy individuals are generally less employable, making it more difficult for them to resist the personal financial risks resulting from work suspension. Such factors can affect the emotions of patients, and emotional fluctuations subsequently exacerbate the disease. This has been confirmed in the patients with psoriasis (Kuang et al., 2020). In this study, the work, income, and changes of the disease condition of patients with SSc affected their emotions. In the initial stages of the pandemic, both patients with SSc and healthy individuals were emotionally depressed due to the unknown risks of the pandemic. However, the ensuing income, illness, and other factors further affected the patients and prevented their emotions from returning to its normal state. At the time of the survey, most healthy individuals had returned to their normal emotional state. Issues with work and income no longer impacted the mood of most healthy people. The factors that affect the emotions of people with SSc are more complex. In addition, because of the rarity of patients with SSc, these patients tend to have less access to resources when seeking medical help. This was demonstrated by the results in our study that showed that patients with SSc received less telemedicine than those with Crohn’s disease (Chen et al., 2020). This is possibly due to fewer medical resources for SSc than for Crohn’s disease treatment. This may be one of the reasons for the higher rates of depression and anxiety among this population compared with the population with inflammation bowel disease (Cheema et al., 2021) and systemic lupus erythematosus (Kasturi et al., 2021).

Traditional Chinese Medicine (TCM) constitution is a unique expression of quality of life, which is essentially the collection of self-conscious symptoms of the whole body of an individual. The TCM constitution theory classifies them into nine categories. This theory creates a link between the physical characteristics of the human body, quality of life, and the diagnosis of TCM syndromes. There are different TCM constitution characteristics for different chronic diseases (Liang et al., 2020). This study confirmed that Yang-deficiency, Qi-deficiency, and blood-stasis constitutions are the most common constitutions of patients with SSc, providing syndromic evidence for TCM intervention in SSc. The results imply that symptoms of cold fear, fatigue, shortness of breath, and reduced microvascular circulation are common in SSc (Supplementary Table 5). Yang deficiency, a constitution characterized by low cold tolerance (Wang and Yao, 2008), is the most prevalent in the SSc population. The patients with SSc have been shown to have low cold tolerance (Cutolo et al., 2010; Herrick et al., 2021). Cold exposure and emotional stress may exacerbate skin fibrosis in SSc (Uehara et al., 2016). High levels of phytoandrogens have been found in traditional Chinese herbs with Yang-tonifying effects (Edouard et al., 2014), which also enhance myocardial ATP-generation capacity (Ko et al., 2006). This result suggests that related herbs, molecules and targets have potential for drug development in SSc. TCM such as Tai Chi (Cetin et al., 2020), Gui-Zhi-Fu-Ling pill (Wang et al., 2019), and Yi-Qi-Huo-Xue recipe (Wu et al., 2014) may help to improve SSc. Tai Chi, especially, has been shown to improve biased constitutions including Qi-deficiency and Yang-deficiency (Yu, 2016). Gui-Zhi-Fu-Ling pill is a classic prescription in TCM for treating blood stasis (Matsumoto et al., 2008; Liu et al., 2021). Yi-Qi-Huo-Xue recipe is a unique prescription for Qi-deficiency and blood stasis (Wu et al., 2014). The relationship between TCM constitution and age-related cognitive decline has been demonstrated in a larger sample of elderly people (Sun et al., 2022). Evidence already exists that TCM constitution is related to emotions. Yang-deficiency and Yin-deficiency constitutions in the female population were associated with a higher risk of depression. In contrast, in a college student population, Qi-stagnation and balanced constitutions were significantly associated with depression (Yap et al., 2021), which is similar to our findings. For depression and other emotion problems, herbal formulas have also been developed in TCM, and these formulas target the BDNF-TrkB, MAPK pathway, etc., (He et al., 2022). Our study suggests that patients with SSc who have Qi-deficiency and Yang-deficiency constitutions may need psychological and emotional support.

We explored the correlation between care patterns, TCM constitution factors, and depression-anxiety occurrence during the COVID-19 pandemic in patients with SSc and reached some conclusions. First, we found that patients from different sources differed in a number of factors. A higher percentage of patients in the online group had a lower income than that of those in the hospital group, which may be due to deviations caused by being in a small or medium-sized city, a rural area, and different types of work. The hospital group obtained lower anxiety and depression scores than those obtained by the online group, possibly because the hospital group had access to constant medical care. The underlying reasons for this need to be further explored. Phlegm-dampness constitution was more common in the hospital group, probably because SSc-related lung disease shows similar characteristics to phlegm-dampness constitution. Furthermore, we found that remote work during the outbreak and disease progression was both correlates of depression while oral medications from patient organizations were associated with suicidal ideation in the hospital group. These conclusions may not only explain the impact of different factors on anxiety and depression but also provide a direction for Chinese medical institutions to work hard within this pandemic period. For patients with chronic non-communicable diseases such as SSc, more active psychological interventions from multiple clinical patterns can be provided (Khaustova et al., 2022). We found that some TCM constitutions were related to anxiety or depression. It is noteworthy that in China, TCM has been welcomed by Chinese patients with SSc. The conclusion of this study helps us to judge whether we can use complementary and alternative medicine to screen for anxiety and depression in Chinese patients with SSc. This may make it easier to judge patients at high risk of anxiety and depression and those who need more attention. These results are meaningful for patients with SSc as they are a rare group.

This study has some limitation. We did not quantitatively investigate SSc disease progression in patients and their household economic income. These two factors may have a more significant impact on depression and anxiety in these patients. Similarly, the size of the hospital group was obviously smaller than that of the online group, which may have led to bias in the results. Considering the fact that patients’ compliance and hand involvement in SSc may limit completion of the patient-reported outcome (Frech et al., 2021), we limited the total number of questions to less than 100. Therefore, we could not evaluate quality of the life, economic status, and accurate disease activity in the patients with SSc, which might have affected the precision of the conclusions to some extent.

## 6. Conclusion

We found that Chinese patients with SSc had a high prevalence of depression and anxiety. The TCM constitution of patients with SSc were mainly Yang-deficiency, Qi-deficiency, and blood-stasis constitutions. The COVID-19 pandemic changed the care patterns of Chinese patients with SSc in terms of work, income, and ways of accessing medical care. Work, income, and disease progression were the factors that affected the mood of patients with SSc. Qi-deficiency and Qi-stagnation constitutions were associated with depression, and Qi-stagnation constitution was associated with anxiety in patients with SSc. The TCM constitution may be one of the factors influencing the emotions and even the quality of life of patients with SSc, and further research is needed.

## Data availability statement

The raw data supporting the conclusions of this article will be made available by the authors, without undue reservation.

## Ethics statement

The studies involving human participants were reviewed and approved by the Ethics Committee of Shanghai TCM-Integrated Hospital, Shanghai University of Traditional Chinese Medicine (approval no. 2020-099-1). Written informed consent for participation was not required for this study in accordance with the national legislation and the institutional requirements.

## Author contributions

QK, L-MC, and J-QZ had the original idea. W-ZT and Y-HZ provided the suggestions of the research. QK, L-MC, W-ZT, and Y-HZ designed the research and questionnaire. QK and L-MC wrote the first draft of the manuscript, designed the initial figures, and contributed equally to this manuscript. Z-HD, Y-ZT, and Y-YZ delivered the questionnaire. QK, L-MC, and Z-HD completed the data statistics. Y-YZ and Y-ZT supported in English language. QK and Z-HD edited the manuscript and provided the corrections. J-QZ and L-MC acquired the funding. J-QZ supervised the process. All authors read and approved the final manuscript.
