# Supplementary figures and images for: Care patterns and Traditional Chinese Medicine constitution as factors of depression and anxiety in patients with systemic sclerosis: A cross-sectional study during the COVID-19 pandemic
Source: Front Integr Neurosci. 2023 Feb 14;17:1052683. doi: 10.3389/fnint.2023.1052683 (PMC9971602; doi:10.3389/fnint.2023.1052683)

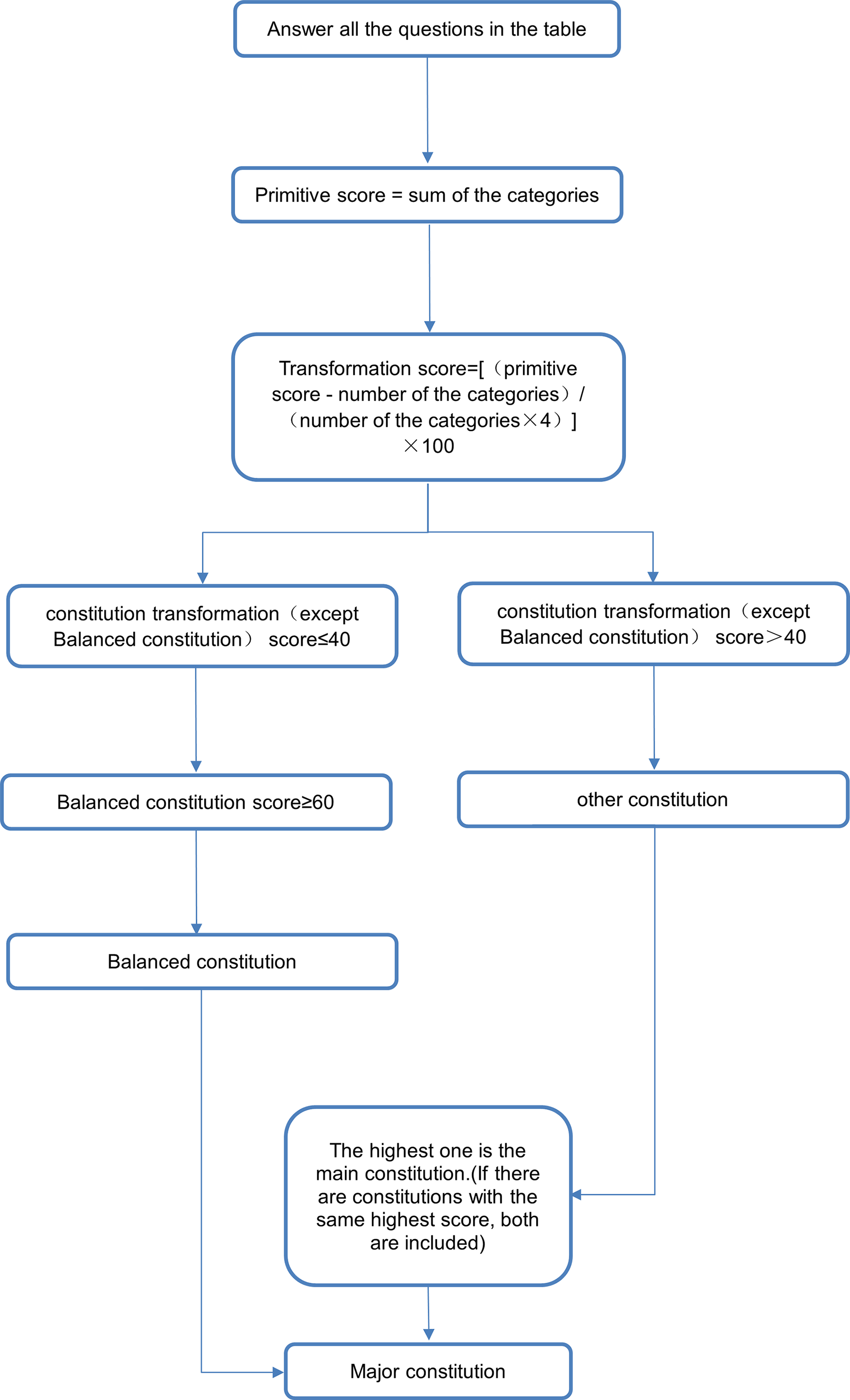

Supplement: Supplementary file 1 [file Image_1.TIF]

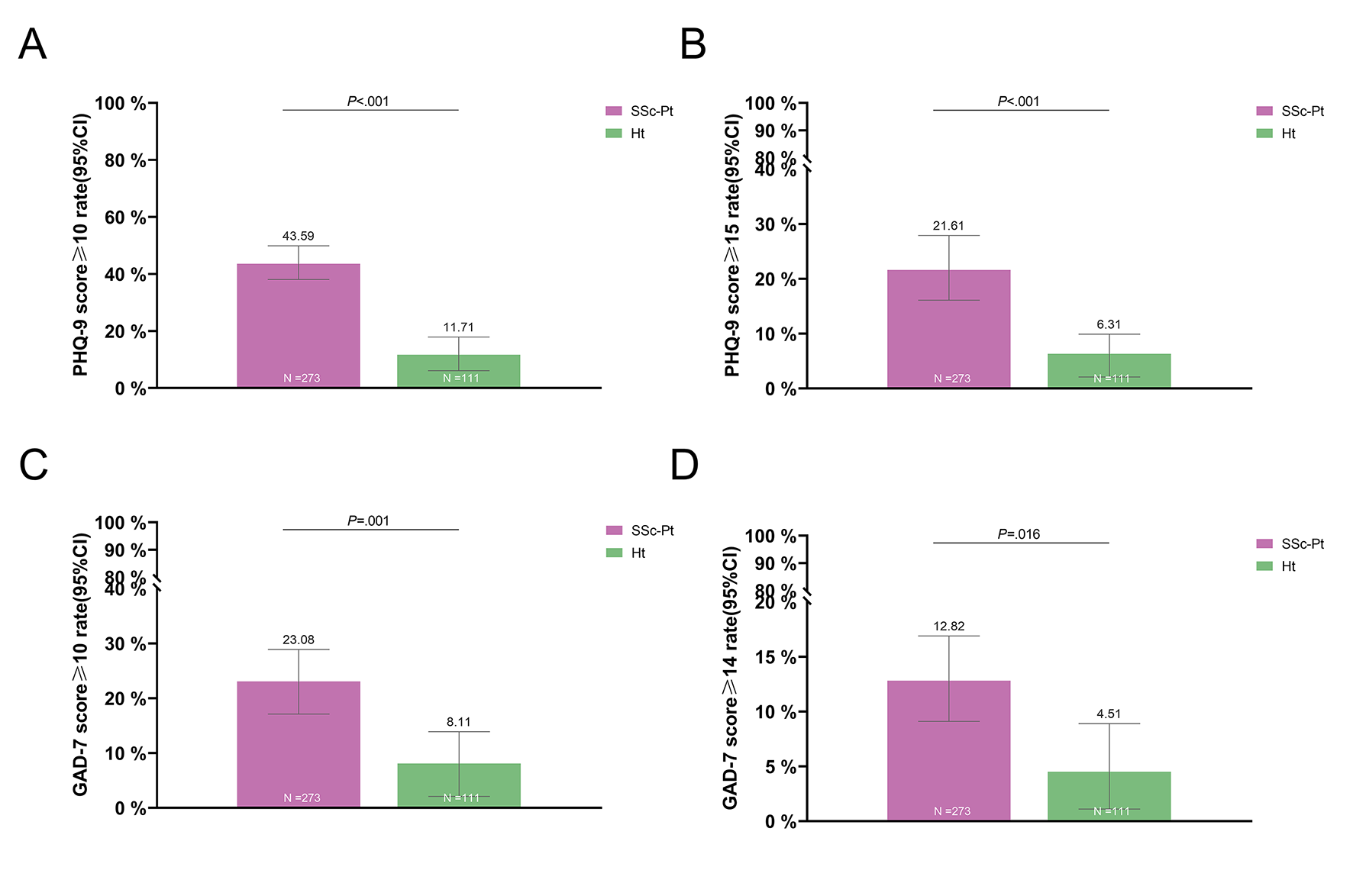

Supplement: Supplementary file 2 [file Image_2.TIF]
