# Supplementary material for: Care patterns and Traditional Chinese Medicine constitution as factors of depression and anxiety in patients with systemic sclerosis: A cross-sectional study during the COVID-19 pandemic
Source: Front Integr Neurosci. 2023 Feb 14;17:1052683. doi: 10.3389/fnint.2023.1052683 (PMC9971602; doi:10.3389/fnint.2023.1052683)

**Supplementary Table 1 English version of care pattern questionnaire**

**Part1 Impact on employment**

1. Working place

a. Same as before

b. Remote work

2. Income

a. Increased

b. No change

c. Reduced

**Part2 Impact on negative emotions (anxiety, stress, sadness) during the outbreak**

3. During early outbreak (Dec 2019 - Feb 2020)

a. Normal status

b. Mild

c. Moderate

d. Severe

4. During mid outbreak (Mar 2020 - May 2020)

a. Normal status

b. Mild

c. Moderate

d. Severe

5. During late outbreak (Jun 2020 - Aug 2020)

a. Normal status

b. Mild

c. Moderate

d. Severe

6. Reasons for emotional change during outbreak

a. Worried about being isolated or becoming a burden to family and society

b. Worried about infection of myself and family

c. Worried about the decline in income for yourself and your family

d. Worried about national and social loss

e. Worried about having difficulties in seeing a doctor

f. Felt emptiness and anxiety

g. Worried about the epidemic making daily life inconvenient

h. Others

i. Not affected

**Part3 Impact on SSc disease progress**

7. Disease progress during the outbreak

a. Improved

b. Stable

c. Aggravated

8. The detail of aggravation in disease progress during the outbreak

a. Aggravated, but was afraid to see a doctor due to the outbreak

b. Aggravated, and had to see a doctor

c. Aggravated, and had to be admitted to the hospital

d. Aggravated, and had to have an surgery

9. Mode of seeing a doctor during the outbreak

a. Not seeing a doctor

b. Used telemedicine

c. Sought help or advice from other patients

d. Go to hospital

10. Ways of telemedicine during the outbreak

a. Treatments and Medications provided by SSc professionals online

b. Free online consultation from non-profitable organizations

c. Online consultation from APPs

d. Online consultation from private doctors and institutions

e. Others

11. Change in SSc medications during the outbreak

a. No change

b. Change in few/most medications

c. Change in all medications

12. Reason for changing medications during the outbreak

a. No access to medications

b. Unable to continue the drug treatment by injection / drip / infusion/

c.Stable disease condition

d. Requested by the doctor

e.Others

13. Sources of oral medications during the outbreak

a. Online drug delivery

b. Hospital pharmacies as before

c. Direct mail from pharmaceutical companies

d. Shared from other patients

e. From patient associations

f. Other ways

g. Not taking oral medications

14. Current disease condition after outbreak

a. Improved

b. Stable

c. Aggravated

15.The detail of aggravation in current disease condition after outbreak

a. Aggravated, but was afraid to see a doctor due to the outbreak

b. Aggravated, and had to see a doctor

c. Aggravated, and had to be admitted to the hospital

d. Aggravated, and had to have an surgery

16. Current and future method of seeing doctors

a. Go to hospital

b. Plan to increase the use of virtual visits sometimes

c. Plan to increase the use of virtual visits most of time

d. Plan to use telemedicine as much as possible

17. Current medications

a. No change

b. Change in few/most medications

c. Change in all medications

18.Current sources of oral medications

a. No change, mainly from hospital pharmacies as before

b. Obtained few medications online

c. Obtained most medications online

d. Obtained all medications online

e. Other ways

**Supplementary Table 2 The result of care patterns**

| Impact on SSc disease progress (n=273/210/63) |  | SSc-Pt,n(%) | SSc-Ol,n(%) | SSc-Hosp,n(%) |
| --- | --- | --- | --- | --- |
| 1. Disease progress during the outbreak | a. Improved | 9(3.30) | 6(2.86) | 3(4.76) |
|  | b. Stable | 163(59.71) | 126(60.00) | 37(58.73) |
|  | c. Aggravated | 101(37.00) | 78(37.14) | 23(36.51) |
| 2. The detail of aggravation in disease progress during the outbreak (n=101/78/23) | a. Aggravated, but was afraid to see a doctor due to the outbreak | 65(64.36) | 54(69.23) | 11(47.83) |
|  | b. Aggravated, and had to see a doctor | 22(21.78) | 16(20.51) | 6(26.09) |
|  | c. Aggravated, and had to be admitted to the hospital | 11(10.89) | 6(7.69) | 5(21.74) |
|  | d. Aggravated, and had to have an surgery | 3(2.97) | 2(2.56) | 1(4.35) |
| 3. Mode of seeing a doctor during the outbreak | a. Not seeing a doctor | 125(45.79) | 100(47.62) | 25(39.68) |
|  | b. Used telemedicine | 34(12.45) | 30(14.29) | 4(6.35) |
|  | c. Sought help or advice from other patients | 39(14.29) | 31(14.76) | 8(12.70) |
|  | d. Go to hospital | 75(27.47) | 49(23.33) | 26(41.27) |
| 4. Ways of telemedicine during the outbreak (n=34/30/4) | a. Treatments and Medications provided by SSc professionals online | 17(50.00) | 14(46.67) | 3(75.00) |
|  | b. Free online consultation from non-profitable organizations | 3(8.82) | 3(10.00) | 0(0.00) |
|  | c. Online consultation from APPs | 12(35.29) | 11(16.67) | 1(25.00) |
|  | d. Online consultation from private doctors and institutions | 6(17.65) | 6(20.00) | 0(0.00) |
|  | e. Others | 2(5.88) | 2(6.67) | 0(0.00) |
| 5. Change in SSc medications during the outbreak | a. No change | 191(69.96) | 146(69.52) | 45(71.43) |
|  | b. Change in few/most medications | 71(26.01) | 56(26.66) | 15(23.81) |
|  | c. Change in all medications | 11(4.02) | 8(3.81) | 3(4.76) |
| 6. Reason for changing medications during the outbreak (n=82/64/18) | a. No access to medications | 40(48.78) | 30(46.88) | 10(55.56) |
|  | b. Unable to continue the drug treatment by injection / drip / infusion | 8(9.76) | 4(6.25) | 4(22.22) |
|  | c. Stable disease condition | 18(21.95) | 16(25.00) | 2(11.11) |
|  | d. Requested by the doctor | 19(23.17) | 16(25.00) | 3(16.67) |
|  | e. Others | 15(18.29) | 10(15.63) | 5(27.78) |
| 7. Sources of oral medications during the outbreak | a. Online drug delivery | 88(32.23) | 73(34.76) | 15(23.81) |
|  | b. Hospital pharmacies as before | 137(50.18) | 98(46.67) | 39(61.9) |
|  | c. Direct mail from pharmaceutical companies | 7(2.56) | 6(2.86) | 1(1.59) |
|  | d. Shared with other patients | 13(4.76) | 10(4.76) | 3(4.76) |
|  | e. From patient associations | 56(20.51) | 50(23.81) | 6(9.52) |
|  | f. Other ways | 31(11.36) | 21(10.00) | 10(15.87) |
|  | g. Not taking oral medications | 14(5.13) | 10(4.76) | 4(6.35) |
| 8. Current disease condition after outbreak | a. Improved | 21(7.69) | 13(6.19) | 8(12.70) |
|  | b. Stable | 189(69.23) | 147(70.00) | 42(66.67) |
|  | c. Aggravated | 63(23.08) | 50(23.81) | 13(20.63) |
| 9. The detail of aggravation in current disease condition after outbreak  (n=63/50/13) | a. Aggravated, but was afraid to see a doctor due to the outbreak | 21(33.33) | 20(40.00) | 1(7.69) |
|  | b. Aggravated, and had to see a doctor | 28(44.44) | 20(40.00) | 8(61.54) |
|  | c. Aggravated, and had to be admitted to the hospital | 12(19.05) | 9(18.00) | 3(23.08) |
|  | d. Aggravated, and had to have an surgery | 2(3.17) | 1(2.00) | 1(7.69) |
| 10. Current and future method of seeing doctors | a. Go to hospital | 109(39.93) | 76(36.19) | 33(52.38) |
|  | b. Plan to increase the use of virtual visits sometimes | 45(16.48) | 37(17.62) | 8(12.70) |
|  | c. Plan to increase the use of virtual visits most of time | 86(31.50) | 67(31.90) | 19(30.16) |
|  | d. Plan to use telemedicine as much as possible | 33(12.09) | 30(14.29) | 3(4.76) |
| 11. Current medications | a. No change | 153(56.04) | 118(56.19) | 35(55.56) |
|  | b. Change in few/most medications | 115(42.12) | 88(41.90) | 27(42.86) |
|  | c. Change in all medications | 5(1.83) | 4(1.90) | 1(1.59) |
| 12. Current sources of oral medications | a. No change, mainly from hospital pharmacies as before | 154(56.41) | 112(53.33) | 42(66.67) |
|  | b. Obtained few medications online | 69(25.27) | 52(24.76) | 17(26.98) |
|  | c. Obtained most medications online | 33(12.09) | 30(14.29) | 3(4.76) |
|  | d. Obtained all medications online | 6(2.20) | 6(2.86) | 0(0.00) |
|  | e. Other ways | 11(4.03) | 10(4.76) | 1(1.59) |

SSc-Pt: Systemic sclerosis patients; SSc-Ol: Online systemic sclerosis patients; SSc-Hosp: Hospital systemic sclerosis patients

**Supplementary Table 3 The detail of TCM constitution**

Supplementary Table 3A TCM constitution

|  | QDC | YADC | YIDC | PDC | DHC | BSC | QSC | ISC | BC |
| --- | --- | --- | --- | --- | --- | --- | --- | --- | --- |
| SSc-Ol,n(%)  (n=210) | 160(76.19) | 177(84.29) | 92(43.81) | 125(59.52) | 84(40.00) | 123(58.57) | 121(57.62) | 62(29.52) | 3(1.43) |
| SSc-Hosp,n(%)  (n=63) | 41(65.08) | 47(74.60) | 23(36.51) | 27(42.86) | 17(26.98) | 33(52.38) | 23(36.51) | 10(15.87) | 3(4.76) |
| Ht,n(%)  (n=111) | 37(33.33) | 23(20.72) | 26(23.42) | 26(23.42) | 20(18.02) | 24(21.62) | 28(25.23) | 15(13.51) | 48(43.24) |

Supplementary Table 3B Major TCM constitution

|  | QDC-major | YADC-major | YIDC-major | PDC-major | DHC-major | BSC-major | QSC-major | ISC-major | BC-major |
| --- | --- | --- | --- | --- | --- | --- | --- | --- | --- |
| SSc-Ol,n(%)  (n=210) | 26(12.38) | 116(55.24) | 4(1.90) | 10(4.76) | 8(3.81) | 25(11.90) | 20(9.52) | 5(2.38) | 3(1.43) |
| SSc-Hosp,n(%)  (n=63) | 9(14.29) | 38(60.32) | 3(4.76) | 1(1.59) | 1(1.59) | 6(9.52) | 1(1.59) | 3(4.76) | 3(4.76) |
| Ht,n(%)  (n=111) | 14(12.61) | 12(10.81) | 3(2.70) | 6(5.41) | 5(4.50) | 10(9.01) | 9(8.11) | 6(5.41) | 48(43.24) |

Supplementary Table 3C Rate of over 2 TCM constitutions

|  | TCM constitution>2 |
| --- | --- |
| SSc-Ol,n(%)  (n=210) | 158(75.24) |
| SSc-Hosp,n(%)  (n=63) | 36(57.14) |
| Ht,n(%)  (n=111) | 32(27.03) |

SSc-Ol, Online systemic sclerosis patients; SSc-Hosp, Hospital systemic sclerosis patients; Ht, Healthy person; BC, Balanced constitution; YADC, Yang-deficiency constitution; YIDC, Yin-deficiency constitution; QDC, Qi-deficiency constitution; QSC:Qi-stagnation constitution; PDC, Phlegm-dampness constitution; DHC, Dampness-heat constitution; BSC, Blood-stasis constitution; ISC, Inherited special constitution; TCM Constitution, Traditional Chinese medicine constitution

**Supplementary Table 4 Complete table of table 2**

**Supplementary Table 4A Depression**

| Source | Question | Item | P | OR（95% CI） | Adjusted P | Adjusted OR（95% CI） |
| --- | --- | --- | --- | --- | --- | --- |
| SSc-Pt |  | Qi-stagnation constitution | 0.000 | 7.978(4.092,15.551) | 0.000 | 3.824(1.799,8.126) |
| SSc-Pt |  | Qi-deficiency constitution | 0.000 | 6.481(3.553,11.823) | 0.025 | 2.250(1.109,4.562) |
| SSc-Pt |  | Dampness-heat constitution | 0.000 | 3.355(1.728,6.513) | 0.898 | 1.058(0.444,2.526) |
| SSc-Pt |  | Phlegm-dampness constitution | 0.000 | 4.648(2.568,8.413) | 0.454 | 1.351(0.614,2.971) |
| SSc-Pt |  | Inherited special constitution | 0.002 | 3.568(1.614,7.891) | 0.534 | 1.346(0.528,3.431) |
| SSc-Pt |  | Blood-stasis constitution | 0.000 | 3.275(1.857,5.775) | 0.468 | 1.295(0.644,2.602) |
| SSc-Pt |  | Yang-deficiency constitution | 0.000 | 3.331(1.745,6.360) | 0.604 | 1.227(0.566,2.658) |
| SSc-Pt |  | Yin-deficiency constitution | 0.000 | 4.978(2.523,9.824) | 0.120 | 1.996(0.835,4.775) |
| SSc-Pt |  | Dampness-heat constitution(main) | 0.011 | 0.160(0.039,0.658) | 0.011 | 0.160(0.039,0.658) |
| SSc-Pt | 1 | Change in working condition during the outbreak(Dec 2019 - Feb 2020) | 0.004 | 2.308(1.309,4.070) | 0.041 | 1.920(1.027,3.589) |
| SSc-Pt | 2 | Income changed during the outbreak(Dec 2019 - Feb 2020) | 0.015 | - | 0.151 | - |
|  | 2B | No change(#dummy variable) | - | - | - | - |
|  | 2A | Increased | 0.087 | 0.219(0.039,1.247) | 0.236 | 0.236(0.307,0.043) |
|  | 2C | Reduced | 0.038 | 1.818(1.034,3.195) | 0.172 | 0.172(1.539,0.829) |
| SSc-Pt | 7 | Disease progress during the outbreak(Dec 2019 - Feb 2020) | 0.000 | - | 0.014 | - |
|  | 7B | Stable(#dummy variable) | - | - | - | - |
|  | 7A | Improved | 0.565 | 0.672(0.174,2.602) | 0.743 | 0.761(0.149,3.888) |
|  | 7C | Aggravated | 0.000 | 5.497(2.580,11.712) | 0.005 | 3.556(1.466,8.626) |
| SSc-Pt | 11 | Change in SSc medications during the outbreak(Dec 2019 - Feb 2020) | 0.013 | - | 0.277 | - |
|  | 11A | No change(#dummy variable) | - | - | - | - |
|  | 11B | Change in few/most medications | 0.004 | 3.079(1.435,6.607) | 0.151 | 1.866(0.797,4.370) |
|  | 11C | Change in/discontinuation of all medications | 0.381 | 2.011(0.422,9.597) | 0.414 | 1.983(0.384,10.230) |
| SSc-Pt | 13E | From patient associations | 0.033 | 2.400(1.074,5.365) | 0.168 | 1.839(0.773,4.376) |
| SSc-Pt | 14 | Current disease status | 0.006 | - | 0.473 | - |
|  | 14B | Stable(#dummy variable) | - | - | - | - |
|  | 14A | Improved | 0.486 | 1.452(0.508,4.151) | 0.866 | 0.890(0.229,3.455) |
|  | 14C | Aggravated | 0.001 | 4.312(1.760,10.559) | 0.239 | 1.886(0.655,5.430) |
| SSc-Ol |  | Qi-deficiency constitution | 0.000 | 6.110(2.974,12.552) | 0.046 | 2.421(1.016,5.767) |
| SSc-Ol |  | Yang-deficiency constitution | 0.008 | 2.945(1.328,6.531) | 0.783 | 1.143(0.442,2.953) |
| SSc-Ol |  | Yin-deficiency constitution | 0.001 | 4.049(1.835,8.936) | 0.211 | 1.950(0.685,5.553) |
| SSc-Ol |  | Phlegm-dampness constitution | 0.003 | 2.767(1.407,5.444) | 0.536 | 0.744(0.293,1.894) |
| SSc-Ol |  | Dampness-heat constitution | 0.042 | 2.129(1.027,4.413) | 0.812 | 0.890(0.341,2.324) |
| SSc-Ol |  | Blood-stasis constitution | 0.005 | 2.625(1.336,5.158) | 0.757 | 1.142(0.493,2.644) |
| SSc-Ol |  | Qi-stagnation constitution | 0.000 | 8.453(3.797,18.816) | 0.000 | 5.151(2.114,12.551) |
| SSc-Ol |  | Inherited special constitution | 0.010 | 3.339(1.334,8.362) | 0.315 | 1.736(0.592,5.090) |
| SSc-Ol |  | Dampness-heat constitution(main) | 0.011 | 0.148(0.034,0.646) | 0.011 | 0.148(0.034,0.646) |
| SSc-Ol | 7 | Disease progress during the outbreak(Dec 2019 - Feb 2020) | 0.001 | - | 0.052 | - |
|  | 7B | Stable(#dummy variable) | - | - | - | - |
|  | 7A | Improved | 0.902 | 0.897(0.158,5.102) | 0.786 | 0.768(0.114,5.167) |
|  | 7C | Aggravated | 0.000 | 8.293(2.831,24.291) | 0.017 | 4.507(1.311,15.492) |
| SSc-Ol | 11 | Change in SSc medications during the outbreak(Dec 2019 - Feb 2020) | 0.059 | - | 0.312 | - |
|  | 11A | No change(#dummy variable) | - | - | - | - |
|  | 11B | Change in few/most medications | 0.022 | 2.932(1.164,7.387) | 0.173 | 1.970(0.742,5.232) |
|  | 11C | Change in/discontinuation of all medications | 0.406 | 2.463(0.293,20.677) | 0.433 | 2.401(0.269,21.457) |
| SSc-Ol | 14 | Current disease status | 0.008 | - | 0.398 | - |
|  | 14B | Stable(#dummy variable) | - | - | - | - |
|  | 14A | Improved | 0.340 | 2.127(0.452,10.015) | 0.551 | 1.697(0.298,9.659) |
|  | 14C | Aggravated | 0.003 | 9.283(2.157,39.960) | 0.208 | 2.942(0.549,15.782) |
| SSc-Hosp |  | Qi-deficiency constitution | 0.001 | 6.643(2.112,20.892) | 0.143 | 3.002(0.691,13.047) |
| SSc-Hosp |  | Yang-deficiency constitution | 0.036 | 3.556(1.089,11.611) | 0.979 | 1.021(0.220,4.734) |
| SSc-Hosp |  | Yin-deficiency constitution | 0.003 | 8.148(2.083,31.875) | 0.405 | 2.102(0.365,12.103) |
| SSc-Hosp |  | Phlegm-dampness constitution | 0.000 | 22.115(4.497,108.749) | 0.032 | 7.537(1.187,47.851) |
| SSc-Hosp |  | Dampness-heat constitution | 0.008 | 17.455(2.134,142.748) | 0.296 | 4.295(0.279,66.099) |
| SSc-Hosp |  | Blood-stasis constitution | 0.002 | 5.571(1.838,16.890) | 0.415 | 1.814(0.434,7.582) |
| SSc-Hosp |  | Qi-stagnation constitution | 0.009 | 5.250(1.513,18.216) | 0.758 | 0.744(0.113,4.885) |
| SSc-Hosp | 1 | Change in working condition during the outbreak(Dec 2019 - Feb 2020) | 0.005 | 4.833(1.617,14.451) | 0.007 | 4.873(1.545,15.371) |
| SSc-Hosp | 7 | Disease progress during the outbreak(Dec 2019 - Feb 2020) | 0.085 | - | 0.112 | - |
|  | 7B | Stable(#dummy variable) | - | - | - | - |
|  | 7A | Improved | 0.556 | 0.474(0.039,5.688) | 0.451 | 0.365(0.027,5.019) |
|  | 7C | Aggravated | 0.042 | 3.411(1.046,11.122) | 0.068 | 3.232(0.918,11.380) |
| Ht |  | Qi-deficiency constitution | 0.000 | 5.111(2.183,11.965) | 0.473 | 1.584(0.451,5.567) |
| Ht |  | Yang-deficiency constitution | 0.001 | 5.76(2.115,15.685) | 0.296 | 1.897(0.572,6.294) |
| Ht |  | Yin-deficiency constitution | 0.001 | 4.801(1.883,12.239) | 0.702 | 1.312(0.327,5.268) |
| Ht |  | Phlegm-dampness constitution | 0.014 | 3.094(1.252,7.646) | 0.252 | 0.434(0.104,1.813) |
| Ht |  | Dampness-heat constitution | 0.002 | 5.531(1.922,15.914) | 0.143 | 2.957(0.693,12.619) |
| Ht |  | Blood-stasis constitution | 0.017 | 3.111(1.228,7.885) | 0.654 | 0.736(0.192,2.818) |
| Ht |  | Qi-stagnation constitution | 0.000 | 7.381(2.833,19.232) | 0.076 | 3.132(0.887,11.068) |
| Ht |  | Balanced constitution | 0.000 | 0.146(0.057,0.374) | 0.131 | 0.381(0.109,1.334) |
| Ht |  | Balanced constitution（main） | 0.000 | 0.146(0.057,0.374) | 0.000 | 0.171(0.065,0.447) |
| Ht |  | Qi-stagnation constitution（main） | 0.019 | 7.00(1.379,35.531) | 0.139 | 3.500(0.666,18.399) |

**Supplementary Table 4B Anxiety**

| Source | Question | Item | P | OR（95% CI） | Adjusted P | Adjusted OR（95% CI） |
| --- | --- | --- | --- | --- | --- | --- |
| SSc-Pt |  | Qi-deficiency constitution | 0.000 | 3.932(2.184,7.080) | 0.245 | 1.538(0.744,3.182) |
| SSc-Pt |  | Yang-deficiency constitution | 0.004 | 2.604(1.356,5.002) | 0.723 | 1.150(0.532,2.484) |
| SSc-Pt |  | Yin-deficiency constitution | 0.005 | 2.029(1.243,3.311) | 0.729 | 0.884(0.438,1.780) |
| SSc-Pt |  | Phlegm-dampness constitution | 0.000 | 2.895(1.766,4.747) | 0.464 | 1.284(0.658,2.503) |
| SSc-Pt |  | Dampness-heat constitution | 0.007 | 1.998(1.209,3.301) | 0.955 | 1.020(0.516,2.014) |
| SSc-Pt |  | Blood-stasis constitution | 0.000 | 2.404(1.471,3.927) | 0.375 | 1.319(0.715,2.432) |
| SSc-Pt |  | Qi-stagnation constitution | 0.000 | 6.465(3.814,10.959) | 0.000 | 4.567(2.504,8.328) |
| SSc-Pt |  | Inherited special constitution | 0.008 | 2.140(1.224,3.742) | 0.712 | 1.133(0.583,2.205) |
| SSc-Pt |  | Qi-stagnation constitution（main） | 0.025 | 3.251(1.156,9.144) | 0.025 | 3.251(1.156,9.144) |
| SSc-Pt | 7 | Disease progress during the outbreak(Dec 2019 - Feb 2020) | 0.003 | - | 0.258 | - |
|  | 7B | Stable(#dummy variable) | - | - | - | - |
|  | 7A | Improved | 0.191 | 0.344(0.069,1.704) | 0.160 | 0.261(0.040,1.704) |
|  | 7C | Aggravated | 0.003 | 2.172(1.303,3.619) | 0.530 | 1.236(0.638,2.393) |
| SSc-Pt | 11 | Change in SSc medications during the outbreak(Dec 2019 - Feb 2020) | 0.000 | - | 0.006 | - |
|  | 11A | No change(#dummy variable) | - | - |  | - |
|  | 11B | Change in few/most medications | 0.000 | 3.717(2.042,6.763) | 0.002 | 2.884(1.452,5.729) |
|  | 11C | Change in/discontinuation of all medications | 0.063 | 3.621(0.932,14.076) | 0.192 | 2.608(0.619,10.991) |
| SSc-Pt | 13D | Shared from other patients | 0.029 | 5.500(1.196,25.302) | 0.059 | 5.609(0.934,33.699) |
| SSc-Pt | 14 | Current disease status | 0.017 | - | 0.214 | - |
|  | 14B | Stable(#dummy variable) | - | - | - | - |
|  | 14A | Improved | 0.336 | 1.563(0.629,3.885) | 0.224 | 2.056(0.644,6.562) |
|  | 14C | Aggravated | 0.005 | 2.345(1.291,4.259) | 0.137 | 1.776(0.833,3.786) |
| SSc-Pt | 17 | Change in current medications | 0.063 | - | 0.883 | - |
|  | 17A | No change(#dummy variable) | - | - | - | - |
|  | 17B | Change in few/most medications | 0.043 | 1.655(1.016,2.697) | 0.954 | 0.983(0.556,1.741) |
|  | 17C | Change in all medications | 0.168 | 4.743(0.518,43.419) | 0.626 | 1.796(0.170,18.983) |
| SSc-Pt | 18 | Current sources of oral  medications | 0.086 | - | 0.390 | - |
|  | 18A | No change, mainly from hospital pharmacies as before(#dummy variable) | - | - | - | - |
|  | 18B | Obtained few medications online | 0.021 | 1.985(1.110,3.549) | 0.432 | 1.293(0.680,2.458) |
|  | 18C | Obtained most medications online | 0.344 | 1.440(0.677,3.064) | 0.632 | 1.222(0.538,2.778) |
|  | 18D | Obtained all medications online | 0.562 | 0.600(0.107,3.373) | 0.197 | 0.269(0.037,1.978) |
|  | 18E | Other ways | 0.095 | 3.200(0.818,12.521) | 0.206 | 2.585(0.593,11.274) |
| SSc-Ol |  | Qi-deficiency constitution | 0.000 | 3.787(1.911,7.504) | 0.302 | 1.566(0.668,3.675) |
| SSc-Ol |  | Yang-deficiency constitution | 0.027 | 2.380(1.103,5.137) | 0.879 | 1.073(0.432,2.667) |
| SSc-Ol |  | Yin-deficiency constitution | 0.025 | 1.889(1.083,3.295) | 0.577 | 0.812(0.390,1.689) |
| SSc-Ol |  | Blood-stasis constitution | 0.004 | 2.262(1.292,3.962) | 0.423 | 1.330(0.662,2.671) |
| SSc-Ol |  | Qi-stagnation constitution | 0.000 | 6.462(3.520,11.860) | 0.000 | 4.885(2.470,9.660) |
| SSc-Ol |  | Phlegm-dampness constitution | 0.001 | 2.667(1.512,4.702) | 0.415 | 1.352(0.655,2.787) |
| SSc-Ol | 7 | Disease progress during the outbreak(Dec 2019 - Feb 2020) | 0.005 | - | 0.175 | - |
|  | 7B | Stable(#dummy variable) | - | - | - | - |
|  | 7A | Improved | 0.172 | 0.220(0.025,1.937) | 0.176 | 0.199(0.019,2.062) |
|  | 7C | Aggravated | 0.005 | 2.332(1.292,4.208) | 0.257 | 1.569(0.720,3.418) |
| SSc-Ol | 11 | Change in SSc medications during the outbreak(Dec 2019 - Feb 2020) | 0.002 | - | 0.011 | - |
|  | 11A | No change(#dummy variable) | - | - | - | - |
|  | 11B | Change in few/most medications | 0.001 | 3.223(1.641,6.330) | 0.006 | 2.700(1.335,5.458) |
|  | 11C | Change in/discontinuation of all medications | 0.129 | 3.537(0.691,18.110) | 0.152 | 3.344(0.641,17.444) |
| SSc-Ol | 14 | Current disease status | 0.040 | - | 0.685 | - |
|  | 14B | Stable(#dummy variable) | - | - | - | - |
|  | 14A | Improved | 0.737 | 1.215(0.390,3.790) | 0.737 | 1.259(0.329,4.819) |
|  | 14C | Aggravated | 0.011 | 2.431(1.224,4.826) | 0.395 | 1.477(0.601,3.629) |
| SSc-Hosp |  | Qi-deficiency constitution | 0.022 | 3.937(1.221,12.696) | 0.430 | 1.748(0.436,7.006) |
| SSc-Hosp |  | Qi-stagnation constitution | 0.002 | 6.026(1.952,18.602) | 0.059 | 3.631(0.955,13.813) |
| SSc-Hosp |  | Dampness-heat constitution | 0.038 | 3.437(1.072,11.022) | 0.588 | 1.502(0.344,6.550) |
| SSc-Hosp |  | Phlegm-dampness constitution | 0.025 | 3.306(1.163,9.400) | 0.721 | 1.292(0.317,5.261) |
| SSc-Hosp | 1 | Change in working condition during the outbreak(Dec 2019 - Feb 2020) | 0.008 | 4.918(1.525,15.859) | 0.008 | 5.882(1.581,21.888) |
|  | 11 | Change in SSc medications during the outbreak(Dec 2019 - Feb 2020) | 0.018 | - | 0.020 | - |
|  | 11A | No change(#dummy variable) | - | - | - | - |
|  | 11B | Change in few/most medications | 0.007 | 6.089(1.648,22.497) | 0.007 | 7.348(1.715,31.475) |
|  | 11C | Change in/discontinuation of all medications | 0.240 | 4.429(0.370,52.990) | 0.242 | 5.048(0.335,76.084) |
| Ht |  | Qi-deficiency constitution | 0.000 | 5.625(2.350,13.461) | 0.571 | 1.469(0.388,5.560) |
| Ht |  | Yang-deficiency constitution | 0.000 | 11.019(3.798,31.967) | 0.034 | 3.818(1.104,13.199) |
| Ht |  | Yin-deficiency constitution | 0.000 | 7.031(2.689,18.381) | 0.365 | 1.948(0.460,8.243) |
| Ht |  | Phlegm-dampness constitution | 0.007 | 3.556(1.424,8.879) | 0.262 | 0.416(0.090,1.925) |
| Ht |  | Dampness-heat constitution | 0.000 | 7.778(2.658,22.755) | 0.077 | 4.518(0.850,24.025) |
| Ht |  | Blood-stasis constitution | 0.009 | 3.492(1.368,8.914) | 0.508 | 0.611(0.142,2.631) |
| Ht |  | Qi-stagnation constitution | 0.000 | 6.988(2.733,17.872) | 0.228 | 2.216(0.608,8.076) |
| Ht |  | Inherited special constitution | 0.015 | 4.038(1.309,12.462) | 0.772 | 0.768(0.129,4.565) |
| Ht |  | Balanced constitution | 0.000 | 0.094(0.030,0.292) | 0.111 | 0.306(0.071,1.316) |
| Ht |  | Balanced constitution（main） | 0.000 | 0.094(0.030,0.292) | 0.001 | 0.124(0.037,0.412) |
| Ht |  | Qi-deficiency constitution（main） | 0.008 | 4.915(1.507,16.028) | 0.189 | 2.296(0.665,7.928) |
| Ht |  | Yang-deficiency constitution（main） | 0.043 | 3.550(1.040,12.123) | 0.428 | 1.685(0.463,6.134) |

**Supplementary Table 4C Suicidal ideation**

| Source | Question | Item | P | OR（95% CI） | Adjusted P | Adjusted OR（95% CI） |
| --- | --- | --- | --- | --- | --- | --- |
| SSc-Pt |  | Qi-deficiency constitution | 0.000 | 4.732(2.294,9.758) | 0.036 | 2.471(1.061,5.757) |
| SSc-Pt |  | Qi-stagnation constitution | 0.000 | 4.872(2.792,8.502) | 0.000 | 3.676(1.937,6.975) |
| SSc-Pt |  | Dampness-heat constitution | 0.000 | 2.607(1.559,4.361) | 0.082 | 1.810(0.927,3.532) |
| SSc-Pt |  | Phlegm-dampness constitution | 0.023 | 1.811(1.086,3.020) | 0.109 | 0.566(0.282,1.135) |
| SSc-Pt |  | Yin-deficiency constitution | 0.001 | 2.368(1.426,3.930) | 0.504 | 1.260(0.640,2.477) |
| SSc-Pt |  | Qi-stagnation constitution（main） | 0.037 | 2.620(1.062,6.462) | 0.037 | 2.620(1.062,6.462) |
| SSc-Pt | 2 | Income changed during the outbreak(Dec 2019 - Feb 2020) | 0.055 | - | 0.407 | - |
|  | 2B | No change(#dummy variable) | - | - | - | - |
|  | 2A | Increased | 0.999 | - | 0.999 | - |
|  | 2C | Reduced | 0.016 | 1.868(1.123,3.106) | 0.180 | 1.474(0.836,2.598) |
| SSc-Pt | 7 | Disease progress during the outbreak(Dec 2019 - Feb 2020) | 0.007 | - | 0.871 | - |
|  | 7B | Stable(#dummy variable) | - | - | - | - |
|  | 7A | Improved | 0.670 | 0.705(0.141,3.519) | 0.704 | 0.698(0.109,4.470) |
|  | 7C | Aggravated | 0.002 | 2.235(1.333,3.749) | 0.781 | 1.106(0.544,2.251) |
| SSc-Pt | 9 | Mode of seeing a doctor during the outbreak | 0.084 | - | 0.270 | - |
|  | 9A | Not seeing a doctor(#dummy variable) | - | - | - | - |
|  | 9B | Used telemedicine | 0.898 | 1.055(0.468,2.376) | 0.950 | 1.029(0.418,2.535) |
|  | 9C | Sought help or advice from other patients | 0.012 | 2.573(1.234,5.363) | 0.065 | 2.191(0.953,5.037) |
|  | 9D | Go to hospital | 0.612 | 1.170(0.637,2.149) | 0.356 | 1.390(0.690,2.799) |
| SSc-Pt | 11 | Change in SSc medications during the outbreak(Dec 2019 - Feb 2020) | 0.000 | - | 0.009 | - |
|  | 11A | No change(#dummy variable) | - | - | - | - |
|  | 11B | Change in few/most medications | 0.000 | 3.976(2.245,7.041) | 0.002 | 2.951(1.477,5.895) |
|  | 11C | Change in/discontinuation of all medications | 0.487 | 1.569(0.441,5.583) | 0.635 | 1.436(0.323,6.393) |
| SSc-Pt | 13D | Shared from other patients | 0.016 | 4.398(1.317,14.681) | 0.089 | 3.068(0.844,11.153) |
| SSc-Pt | 13E | From patient associations | 0.028 | 1.955(1.077,3.55) | 0.470 | 1.298(0.640,2.634) |
| SSc-Pt | 14 | Current disease status | 0.005 | - | 0.215 | - |
|  | 14B | Stable(#dummy variable) | - | - | - | - |
|  | 14A | Improved | 0.426 | 1.462(0.574,3.721) | 0.742 | 1.212(0.386,3.809) |
|  | 14C | Aggravated | 0.001 | 2.612(1.456,4.688) | 0.080 | 1.983(0.921,4.269) |
| SSc-Pt | 17 | Change in current medications | 0.023 | - | 0.277 | - |
|  | 17A | No change(#dummy variable) | - | - | - | - |
|  | 17B | Change in few/most medications | 0.037 | 1.719(1.034,2.858) | 0.912 | 1.036(0.553,1.940) |
|  | 17C | Change in all medications | 0.046 | 9.600(1.044,88.278) | 0.109 | 6.927(0.649,73.989) |
| SSc-Ol |  | Qi-deficiency constitution | 0.000 | 4.405(1.945,9.978) | 0.077 | 2.301(0.913,5.803) |
| SSc-Ol |  | Qi-stagnation constitution | 0.000 | 4.284(2.286,8.031) | 0.001 | 3.059(1.537,6.092) |
| SSc-Ol |  | Dampness-heat constitution | 0.013 | 2.050(1.161,3.621) | 0.327 | 1.426(0.701,2.902) |
| SSc-Ol |  | Yin-deficiency constitution | 0.016 | 2.007(1.141,3.529) | 0.969 | 0.986(0.474,2.048) |
| SSc-Ol |  | Qi-stagnation constitution（main） | 0.044 | 2.630(1.025,6.749) | 0.044 | 2.630(1.025,6.749) |
| SSc-Ol | 7 | Disease progress during the outbreak(Dec 2019 - Feb 2020) | 0.053 | - | 0.642 | - |
|  | 7B | Stable(#dummy variable) | - | - | - | - |
|  | 7A | Improved | 0.410 | 0.400(0.045,3.534) | 0.397 | 0.358(0.033,3.845) |
|  | 7C | Aggravated | 0.030 | 1.900(1.066,3.387) | 0.601 | 0.800(0.346,1.848) |
| SSc-Ol | 9 | Mode of seeing a doctor during the outbreak | 0.064 | - | 0.135 | - |
|  | 9A | Not seeing a doctor |  | - |  | - |
|  | 9B | Used telemedicine | 0.891 | 1.062(0.446,2.530) | 0.878 | 1.076(0.422,2.740) |
|  | 9C | Sought help or advice from other patients | 0.011 | 2.942(1.286,6.734) | 0.033 | 2.599(1.081,6.247) |
|  | 9D | Go to hospital | 0.195 | 1.594(0.788,3.225) | 0.153 | 1.755(0.812,3.796) |
| SSc-Ol | 11 | Change in SSc medications during the outbreak(Dec 2019 - Feb 2020) | 0.001 | - | 0.006 | - |
|  | 11A | No change(#dummy variable) | - | - | - | - |
|  | 11B | Change in few/most medications | 0.000 | 3.583(1.885,6.810) | 0.001 | 3.062(1.540,6.087) |
|  | 11C | Change in/discontinuation of all medications | 0.661 | 1.391(0.318,6.076) | 0.642 | 1.432(0.315,6.510) |
| SSc-Ol | 14 | Current disease status | 0.016 | - | 0.118 | - |
|  | 14B | Stable(#dummy variable) | - | - | - | - |
|  | 14A | Improved | 0.670 | 1.289(0.4,4.151) | 0.798 | 1.195(0.305,4.680) |
|  | 14C | Aggravated | 0.004 | 2.625(1.362,5.060) | 0.039 | 2.629(1.049,6.590) |
| SSc-Hosp |  | Qi-deficiency constitution | 0.043 | 5.185(1.057,25.438) | 0.516 | 1.916(0.270,13.598) |
| SSc-Hosp |  | Qi-stagnation constitution | 0.003 | 6.417(1.850,22.261) | 0.172 | 2.906(0.628,13.442) |
| SSc-Hosp |  | Dampness-heat constitution | 0.004 | 6.268(1.802,21.805) | 0.414 | 1.980(0.384,10.202) |
| SSc-Hosp |  | Inherited special constitution | 0.011 | 6.450(1.528,27.228) | 0.139 | 3.376(0.673,16.921) |
| SSc-Hosp |  | Yin-deficiency constitution | 0.016 | 4.359(1.317,14.429) | 0.418 | 1.945(0.389,9.741) |
| SSc-Hosp | 7 | Disease progress during the outbreak(Dec 2019 - Feb 2020) | 0.044 | - | 0.137 | - |
|  | 7B | Stable(#dummy variable) | - | - | - | - |
|  | 7A | Improved | 0.377 | 3.200(0.243,42.183) | 0.153 | 7.277(0.48,110.394) |
|  | 7C | Aggravated | 0.013 | 4.923(1.407,17.221) | 0.086 | 3.543(0.836,15.010) |
| SSc-Hosp | 11 | Change in SSc medications during the outbreak(Dec 2019 - Feb 2020) | 0.022 | - | 0.070 | - |
|  | 11A | No change(#dummy variable) | - | - | - | - |
|  | 11B | Change in few/most medications | 0.006 | 6.204(1.698,22.667) | 0.025 | 5.804(1.245,27.058) |
|  | 11C | Change in/discontinuation of all medications | 0.440 | 2.714(0.216,34.149) | 0.276 | 4.536(0.298,69.024) |
| SSc-Hosp | 13E | From patient associations | 0.029 | 7.500(1.224,45.961) | 0.025 | 12.124(1.375,106.912) |
| Ht |  | Yang-deficiency constitution | 0.001 | 8.853(2.548,30.757) | 0.005 | 6.839(1.801,25.975) |
| Ht |  | Phlegm-dampness constitution | 0.048 | 3.343(1.011,11.054) | 0.777 | 1.238(0.283,5.428) |
| Ht |  | Blood stasis constitution | 0.030 | 3.810(1.142,12.703) | 0.308 | 2.119(0.501,8.963) |
| Ht |  | Yang-deficiency constitution(main) | 0.022 | 5.000(1.255,19.915) | 0.029 | 5.145(1.179,22.456) |
| Ht |  | Dampness-heat constitution(main) | 0.006 | 14.40(2.145,96.667) | 0.008 | 14.852(2.045,107.864) |
| Ht | 2 | Income changed during the outbreak(Dec 2019 - Feb 2020) | 0.012 | - | - | - |
|  | 2B | No change(#dummy variable) | - | - | - | - |
|  | 2A | Increased | 0.011 | 17.500(1.932,158.538) | - | - |
|  | 2C | Reduced | 0.020 | 4.712(1.273,17.433) | - | - |

SSc-Pt, Systemic sclerosis patients; SSc-Ol, Online systemic sclerosis patients;

SSc-Hosp, Hospital systemic sclerosis patients; Ht, Healthy person

**Supplementary Table 5 Nine TCM constitutions and typical features**

| TCM constitutions | Typical features |
| --- | --- |
| Balanced | A neutral or well-balanced constitution is ideal constitution and very rare in society. This type of body typically has good skin complexion, good appetite, and normal bowel movements. The person feels energetic, sleeps well, and is able to adapt to different environments. |
| Qi Deficient | Qi deficient people would have shortness of breath, have a weak voice, feel tired easily, sweat randomly, and easily catch colds and flus. They might also have a poor appetite, loose stools, and a pale face. Their tongue would be pale, and their pulse would be empty. |
| Yang Deficient | A yang deficient person would feel cold in the hands and feet, dislike cold weather and wind, and easily catch colds and flus. This person may have poor sleep, loose stools, spontaneous sweating, and excess clear urine. The tongue would be pale with a white coating on top, and the pulse would be slow, deep, and weak. |
| Yin Deficient | A yin deficient person feels hot in the hands and feet, dislikes hot and dry weather, and usually has a thin physique. They might also experience dry mouth and nose, constipation, and insomnia. They are more vulnerable to sore throat and fever. Their tongue would be red with little or no coating, and their pulse would be thin and rapid. |
| Damp Phlegm | A person of damp phlegm constitution tends to be overweight, feels heavy or sluggish, have an oily face, sweats a lot, have excess throat secretions to spit, and dislikes rain and damp environments. Their tongue would have a sticky coating, and their pulse would be slow and slippery. |
| Damp Heat | A person with damp heat constitution likely has oily skin, acne, bad breath, bitter taste in mouth, dry stools, yellow urine, and yellow tongue coating. They dislike damp and hot environments. Their tongue would have a sticky yellow coating, and their pulse would be rapid and slippery. Person feels hot and has bad skin with acne. |
| Blood Stagnation | A person with the blood stagnation constitution likely has dull and grayish skin, purple lips and nails, spots on face, dark circles under eyes, and body pains. It’s easy for them to get bruises and bleed, and they feel uncomfortable in cold and windy weather. Their tongue would be purple, and their pulse would be wiry and choppy. |
| Qi Stagnation | A person with the qi stagnation constitution likely has a thinner physique, heart palpitations, and insomnia. Their tongue would be purple, and their pulse would be wiry. |
| Inherited special | A person with a special constitution has inborn sensitivities to certain foods, drugs, smells, pollen, or other environmental allergens. They often develop symptoms such as sneezing, runny nose, itchy skin, coughing, and the like. |

**Supplementary Table 6 Constitution in Chinese Medicine Questionnaire (CCMQ)**

| Chinese | English |
| --- | --- |
| （1）您精力充沛吗？ | (1)Were you energetic? |
| （2）您容易疲乏吗？ | (2)Did you get tired easily? |
| （3）您容易气短（呼吸短促，接不上气）吗？ | (3)Did you suffer from shortness of breath? |
| （4）您容易心慌吗？ | (4)Did you get palpitations? |
| （5）您容易头晕或站起时晕眩吗？ | (5)Did you get dizziness easily or become giddy when standing up? |
| （6）您喜欢安静、懒得说话吗？ | (6)Did you prefer quietness and do not like to talk? |
| （7）您说话声音低弱无力吗？ | (7)Did you feel weak when talking? |
| （8）您感到闷闷不乐、情绪低沉吗？ | (8)Did you feel gloomy and depressed? |
| （9）您容易精神紧张、焦虑不安吗？ | (9)Do you get anxious and worried easily? |
| （10）您多愁善感、感情脆弱吗？ | (10)Did you feel sensitive, vulnerable or emotionally upset? |
| （11）您容易感到害怕或受到惊吓吗？ | (11)Were you easily scared or frightened? |
| （12）您胁肋部或乳房胀痛吗？ | (12)Did you experience distention in the underarm or breast? |
| （13）您感到胸闷或腹部胀满吗？ | (13)Did you feel chest or stomach stuffiness? |
| （14）您无缘无故叹气吗？ | (14)Did you sigh for no reason? |
| （15）您感到身体沉重不轻松或不爽快吗？ | (15)Did your body feel heavy or lethargic? |
| （16）您感到手脚心发热吗？ | (16)Do the palms of your hands or soles of your feet feel hot? |
| （17）您手脚发凉吗？ | (17)Did your hands or feet feel cold or clammy? |
| （18）您胃脘部、背部或腰膝部怕冷吗？ | (18)Did you feel cold easily in your abdomen, back, lower back or knees? |
| （19）您感到怕冷、衣服比别人穿得多吗？ | (19)Were you sensitive to cold and tend to wear more clothes than others? |
| （20）您感觉身体、脸上发热吗？ | (20)Did your body and face feel hot? |
| （21）您比一般人耐受不了寒冷（冬天的寒冷或冷空调、电扇等）吗？ | (21)Did you feel more vulnerable to the cold than others (winter cold ness, air conditioners, fans, etc.)? |
| （22）您比别人容易患感冒吗？ | (22)Did you catch colds more easily than others? |
| （23）您没有感冒时也会打喷嚏吗？ | (23)Did you sneeze even when you did not have a cold? |
| （24）您没有感冒时也会鼻塞、流鼻涕吗？ | (24)Did you have runny or stuffy nose even when you did not have a cold? |
| （25）您有因季节变化、温度变化或异味等原因而咳喘的现象吗？ | (25)Did you cough due to seasonal change, temperature change, or unpleasant odor? |
| （26）您活动量稍大就容易出虚汗吗？ | (26)Did you sweat easily when you had a slightly increased physical activity? |
| （27）您容易忘事（健忘）吗？ | (27)Did you forget things easily? |
| （28）您有额部油脂分泌多的现象吗？ | (28) Did you have an excessively oily forehead and/or T-zone? |
| （29）您口唇的颜色比一般人红吗？ | (29)Were your lips redder than others? |
| （30）您容易过敏（对药物、食物、气味、花粉或在季节交替、气候变化时)吗? | (30)Did you have allergies? (E.g. medicine, food, odors, pollen, pet dander, or during seasonal or weather change etc.)? |
| （31）您的皮肤容易起荨麻疹(风团、风疹块、风疙瘩）吗？ | (31)Did your skin get hives/urticaria easily? |
| （32）您的皮肤因过敏出现过紫癜（紫红色瘀点、瘀斑）吗？ | (32)Did your skin have purpura (purple spots, ecchymosis) due to allergies? |
| （33）您的皮肤常在不知不觉中出现青紫瘀斑（皮下出血）吗？ | (33) Did black or purple bruises appear on your skin for no reason? |
| （34）您的皮肤一抓就红，并出现抓痕吗？ | (34)Did you skin turn red and show traces when you scratched it? |
| （35）您皮肤或口唇干吗？ | (35)Did your skin or lips feel dry? |
| （36）您两颧部有细微红丝吗？ | (36)Did you have visible capillary/thread veins on your cheeks? |
| （37）您身体上有哪里疼痛吗？ | (37)Did you feel pain somewhere in your body? |
| （38）您面部两颧潮红或偏红吗？ | (38)Did you get hot flashes? |
| （39）您面部或鼻部有油腻感或者油亮发光吗？ | (39)Did your nose or your face feel greasy, oily, or shiny? |
| （40）您面色晦暗，或容易出现褐斑吗？ | (40)Did you have a dark face or get brown spots easily? |
| （41）您易生痤疮或者疮疖吗？ | (41)Did you get acne or sores easily? |
| （42）您上眼睑比别人肿（上眼睑有轻微隆起的现象）吗？ | (42)Did you have upper eyelid swelling? |
| （43）您容易有黑眼圈吗？ | (43)Did you get dark circles under the eyes easily? |
| （44）您感到眼睛干涩吗？ | (44)Did your eyes feel dry and use eye drops? |
| （45）您口唇颜色偏暗吗？ | (45)Did your lips darker, more blue or purple than usual? |
| （46）您感到口干咽燥，总想喝水吗？ | (46)Did you often feel parched and need to drink water? |
| （47）您咽喉部有异物感且吐之不出、咽之不下吗？ | (47)Did your throat feel strange (i.e. Like something was stuck or there was a lump in your throat)? |
| （48）您感到口苦或嘴里有异味吗？ | (48)Did you have bitterness or a strange taste in your mouth? |
| （49）您嘴里有黏黏的感觉吗？ | (49)Did your mouth feel sticky? |
| （50）您腹部肥满松软吗？ | (50)Was your stomach/belly flabby? |
| （51）您平时痰多，特别是咽喉部总感到有痰堵着吗？ | (51)Did you have lots of phlegm, especially in your throat? |
| （52）您吃（喝）凉的东西会感到不舒服或者怕吃（喝）凉的东西吗？ | (52)Did you feel uncomfortable when you drank or ate something cold, or do you avoid to drinking or eating something cold? |
| （53）您能适应外界自然和社会环境的变化吗？ | (53)Could you adapt yourself to external natural or social environment change? |
| （54）您容易失眠吗？ | (54)Did you suffer from insomnia? |
| （55）您受凉或吃（喝）凉的东西后，容易腹泻（拉肚子）吗？ | (55)Did you easily contract diarrhea when you were exposed to cold or eat (or drink) something cold? |
| （56）您大便黏滞不爽、有解不尽的感觉吗？ | (56)Did you pass sticky stools and/or feel that your bowel movement is incomplete? |
| （57）您容易便秘或大便干燥吗？ | (57)Did you get constipated easily or have dry stools? |
| （58）您舌苔厚腻或有舌苔厚厚的感觉吗？ | (58)Did your tongue have a thick coating? |
| （59）您小便时尿道有发热感、尿色浓（深）吗？ | (59)Did your urethral canal feel hot when you urinated, or did your urine have a dark color? |
| （60）您的阴囊部位潮湿吗？（限男性回答） | (60)Was your scrotum always wet (only for male interviewees)? |
| （60）您带下色黄(白带颜色发黄)吗?(仅限女性受访者） | (60) Was your vaginal discharge yellowish (only for female interviewees)? |

**Supplementary Table 7 Reliability and validity of care pattern questionnaire**

| Item | Type of coefficient | Value |
| --- | --- | --- |
| Total reliability of care pattern questionnaire (38 items including multiple choice questions) | Cronbach's α | 0.817 |
| Reliability outside of emotion ranking items (35 items including multiple choice questions) | Cronbach's α | 0.813 |
| Reliability of emotion ranking items (3 items) | Cronbach's α | 0.814 |
| Criterion validity of emotion items (Item no. 3 early, vs. the rank of PHQ-9) | Pearson's r | 0.288 |
| Criterion validity of emotion items (Item no. 3 early, vs. the rank of GAD-7) | Pearson's r | 0.292 |
| Criterion validity of emotion items (Item no. 4 mid, vs. the rank of PHQ-9) | Pearson's r | 0.318 |
| Criterion validity of emotion items (Item no. 4 mid, vs. the rank of GAD-7) | Pearson's r | 0.302 |
| Criterion validity of emotion items (Item no. 5 late, vs. the rank of PHQ-9) | Pearson's r | 0.405 |
| Criterion validity of emotion items (Item no. 5 late, vs. the rank of GAD-7) | Pearson's r | 0.387 |

**Supplementary Figure1 Process of identifying TCM Constitution**


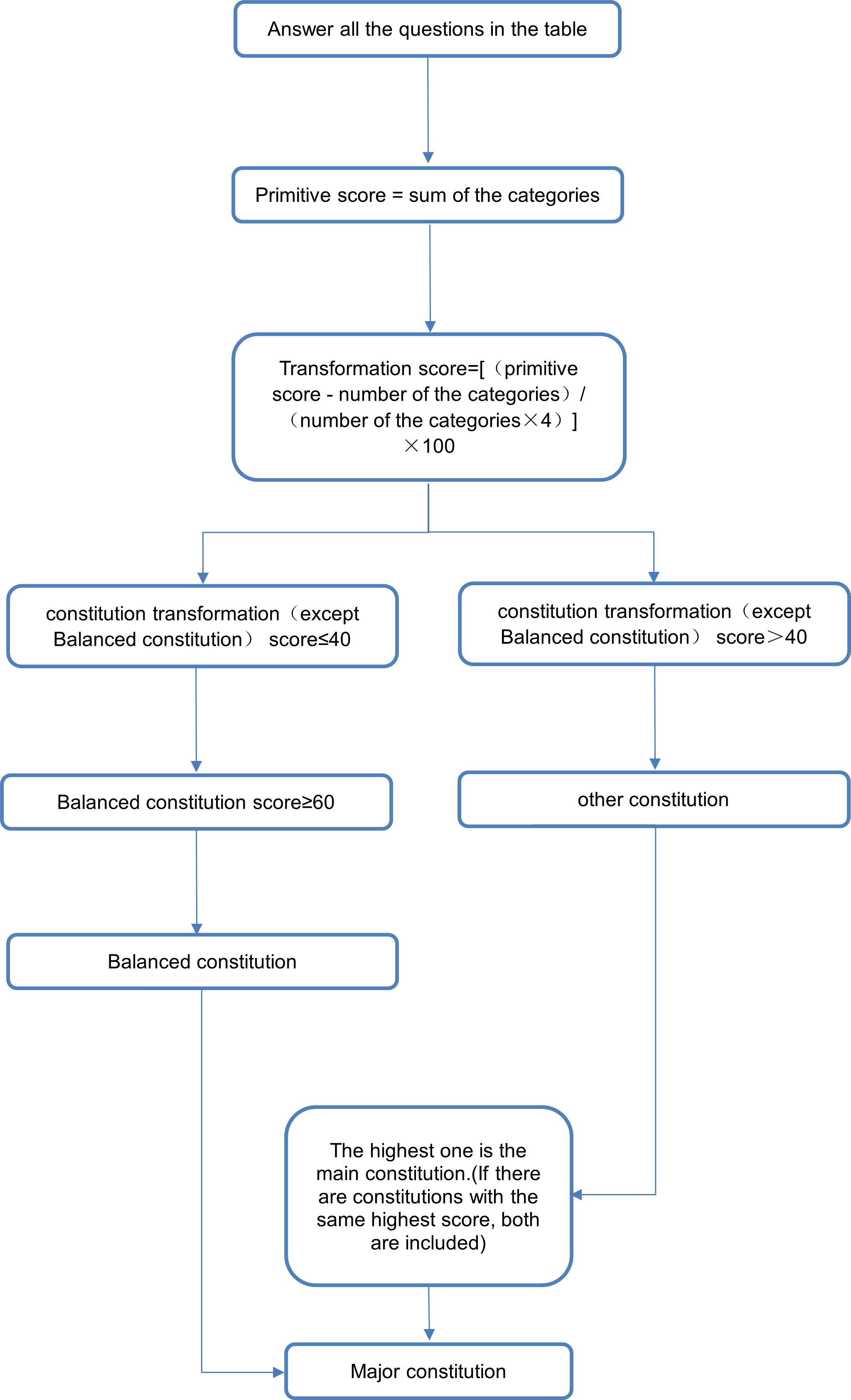


**Supplementary Figure2 Rate of different definition of PHQ-9 and GAD-7**

**(A)** PHQ-9 score ≥10 rate **(B)** PHQ-9 score ≥15 rate **(C)** GAD-7 score ≥10 rate **(D)** GAD-7 score ≥14 rate.

SSc-Pt, Systemic sclerosis patients; SSc-Ol, Online systemic sclerosis patients;

SSc-Hosp, Hospital systemic sclerosis patients; Ht, Healthy person.


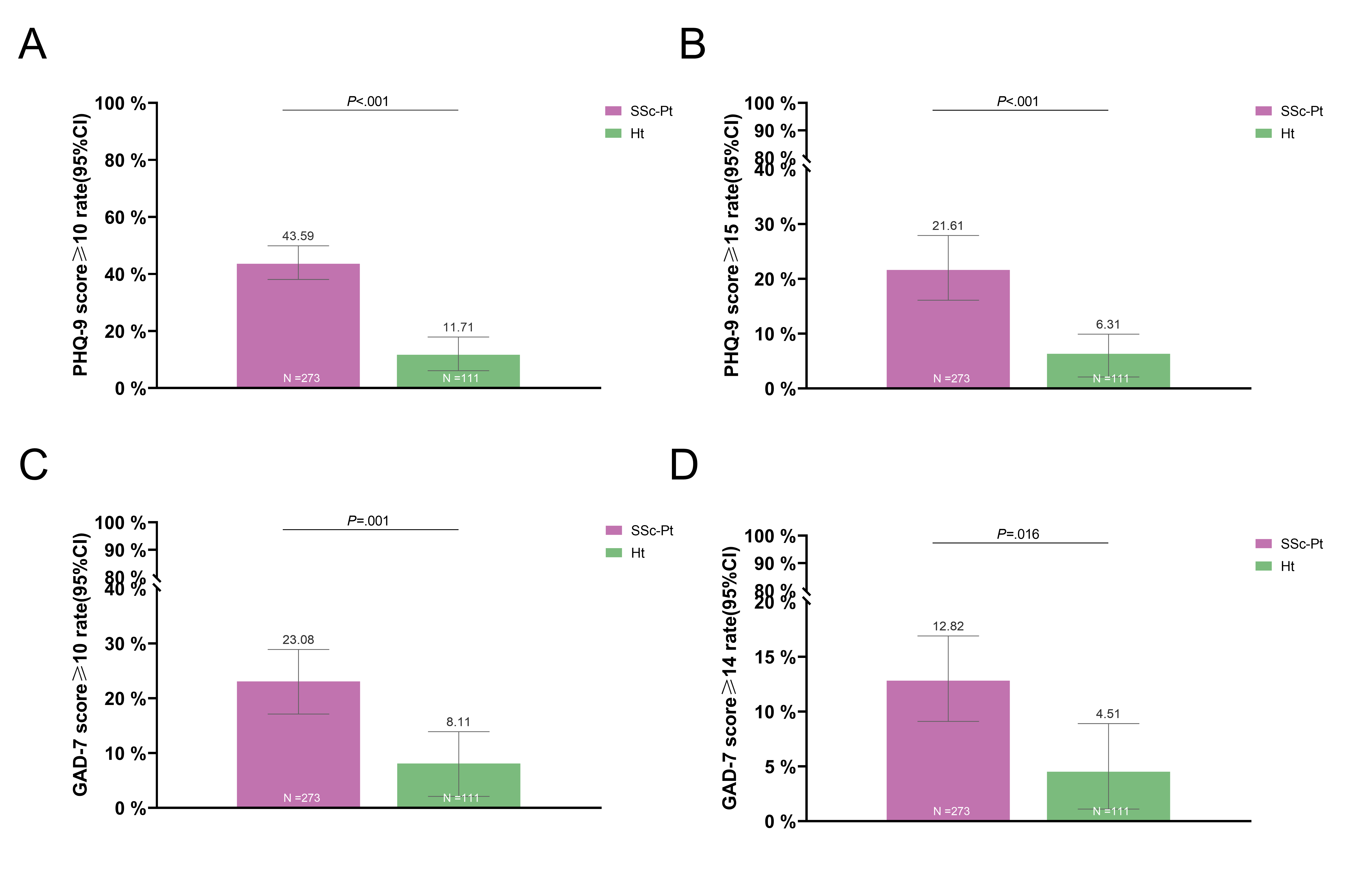

Supplement: Supplementary file 3 [file Data_Sheet_1.docx]
