# Supplementary material for: Care patterns and Traditional Chinese Medicine constitution as factors of depression and anxiety in patients with systemic sclerosis: A cross-sectional study during the COVID-19 pandemic
Source: Front Integr Neurosci. 2023 Feb 14;17:1052683. doi: 10.3389/fnint.2023.1052683 (PMC9971602; doi:10.3389/fnint.2023.1052683)
Supplement: Supplementary file 4 [file Data_Sheet_2.pdf]

上海中医药大学附属上海市中西医结合医院伦理委员会
IRB of Shanghai TCM-Integrated Hospital, Shanghai University of TCM
伦理审查批件
(Approval Notice)

批件号：上海中医药大学附属上海市中西医结合医院伦理委员会2020-099-1

**审查日期**	2020年8月26日	**审查地点**	上海市保定路230号
**项目名称**	系统性硬化症患者横断面研究		
**研究单位**	上海市中西医结合医院	**临床研究批文**	
**主要研究者**	孔琪	**申办者/CRO**	
**审查类别**	初始审查	**审查方式**	会议审查
**审查委员**	张嗣博、周铭、崔燕、董智平、陈颖、盛昭园、张健		
**审查文件**	1 伦理审查申请表<br>2 临床研究方案<br>3 知情同意书<br>4 主要研究者简历<br>5 研究人员名单<br>6 研究问卷		
根据我国国家食品药品监督管理局《药物临床试验质量管理规范》（2010年）国家中医药管理局《中医药临床研究伦理审查管理规范》（2010年）、卫生部《涉及人的生物医学研究伦理审查办法》（2016年）、国家食品药品监督管理局《医疗器械临床试验规定（2004年）》和《药物临床试验质量管理规范》（2003年）的伦理原则，经本伦理委员会审查决定：			√☐同意临床研究<br>☐不同意临床研究<br>☐终止或暂停临床研究
**批件有效期**	2020年8月26日～2021年8月25日	**联系电话**	021-65415910-5223

委员会主席签字：

上海市中西医结合医院 伦理委员会

伦理委员会盖章

日期：2020年8月31日

本审查自签发日期有效期一年，研究负责人必须严格使用经审查同意的知情同意书文本和研究方案。如伦理审查批件失效时不能完成所有的临床研究（包括统计分析），请再本批件失效前一个月，递交跟踪审查申请报告。如研究结束并在审查有效期内，请递交研究结题报告。研究中发生严重不良事件及影响研究风险受益比的非预期不良事件，应立刻报告本伦理委员会；任何研究方案、知情同意书的修改，包括研究人员的变更等，必须递交修正方案伦理审查申请表，经伦理审查委员会审查获得批准后执行。
